# Biodegradation of Synthetic Aliphatic-Aromatic Polyesters in Soils: Linking Chemical Structure to Biodegradability

**DOI:** 10.1021/acs.est.5c03099

**Published:** 2025-09-12

**Authors:** Taylor F. Nelson, Rebekka Baumgartner, Madalina Jaggi, Stefano M. Bernasconi, Glauco Battagliarin, Carsten Sinkel, Andreas Künkel, Hans-Peter E. Kohler, Kristopher McNeill, Michael Sander

**Affiliations:** † Institute of Biogeochemistry and Pollutant Dynamics, 27219 ETH Zurich, Zurich 8092, Switzerland; ‡ Geological Institute, Department of Earth Sciences, 27219 ETH Zurich, Zurich 8092, Switzerland; § 5184 BASF SE, Carl-Bosch-Strasse 38, Ludwigshafen 67056, Germany; ∥ Environmental Biochemistry Group; Environmental Microbiology, Swiss Federal Institute of Aquatic Science and Technology (Eawag), Dübendorf 8600, Switzerland

**Keywords:** biodegradable polyesters, carbon stable isotope labeling, mineralization, soil incubation, structure-biodegradability relationship, polyester extraction

## Abstract

Biodegradable aliphatic-aromatic copolyesters are commercially important and used in diverse applications, including soil-biodegradable mulch films. This work investigates the effects of copolyester chemical structure on soil biodegradability using ^13^C-labeled variants of polybutylene adipate-*co*-terephthalate (PBAT) and polybutylene sebacate-*co*-terephthalate (PBSeT), as well as ^13^C-labeled cellulose as a positive biodegradation control. Biodegradation in soil was assessed by monitoring polyester and cellulose mineralization to ^13^CO_2_ throughout multimonth incubations and quantifying the nonmineralized polyester in the soil after incubations. Mass balances on polyester- and cellulose-added ^13^C over the incubations were closed. The soil biodegradability of PBSeT was higher than that of PBAT for variants with a molar ratio of terephthalate (T) to total diacid of 50%. PBAT biodegradability substantially increased as its T content decreased from 50% to 47, 30, 20, and 0%. Increasing biodegradability with decreasing T content also resulted in preferential biodegradation of aliphatic-rich domains in the initial phase of the incubations. Polyesters undergoing extensive mineralization also showed substantial incorporation of polyester carbon into the soil microbial biomass. Differences in polyester soil biodegradability were rationalized based on differences in their enzymatic hydrolyzability. Qualitative chemical structure-biodegradability relationships can lead to tailoring polyester biodegradability to specific applications and the biodegradation potentials of receiving environments.

## Graphical abstract

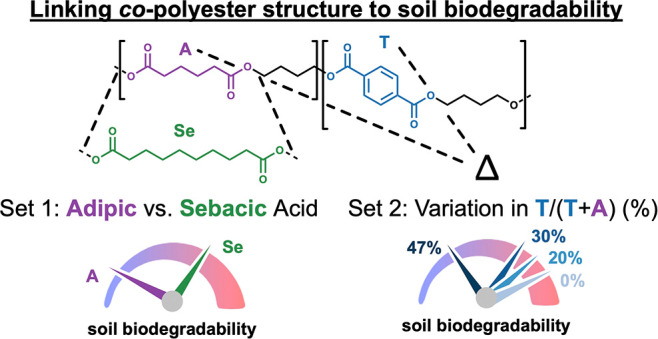

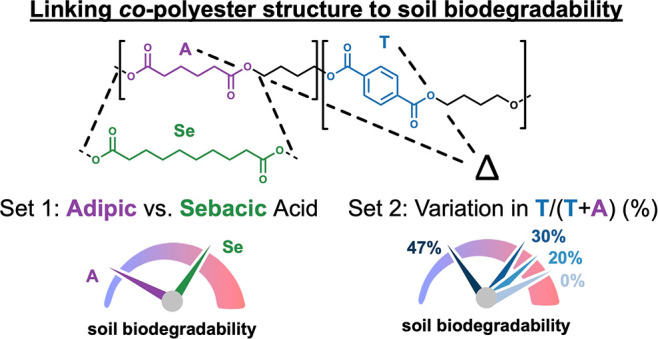

## Introduction

Replacing conventional, environmentally persistent polymers such as polyethylene (PE) with environmentally biodegradable polymers in specific applications is a promising strategy to prevent plastic accumulation in the environment.^1–4^ This is especially true for applications in which plastic products are directly used in the open environment and/or have a high probability of remaining (in part) in the open environment after use. An example is thin (<25 μm thickness) agricultural mulch films: conventional films are PE-based and, if not completely recollected after use, accumulate in agricultural soils over repeated application cycles to concentrations that negatively impact soil productivity.^5–8^ Conversely, mulch films composed of biodegradable polymers undergo biodegradation by soil microorganisms after being plowed into soils after the application period.^8–12^ Under oxic conditions, this process ultimately results in the complete metabolic utilization of polymer carbon to form both CO_2_ (i.e., mineralization) and new microbial biomass. Polymer-derived carbon incorporated into microbial biomass—as part of the soil organic matter pool—also undergoes biodegradation (possibly over longer time frames than the polymer itself), designating CO_2_ as the ultimate end point of biodegradation.^13^ Therefore, biodegradation in soils is typically assessed by soil incubations coupled to respirometric analysis of the formation of polymer-derived CO_2_ over time.^14^

Among the biodegradable polymers with desirable material properties for mulch film applications, and at the same time documented biodegradability in soils, are aliphatic-aromatic copolyesters, including polybutylene adipate-*co*-terephthalate (PBAT)^15^ and polybutylene sebacate-*co*-terephthalate (PBSeT).^16^ As a copolyester synthesized from two aliphatic (i.e., the diol butanediol (B) and either the diacid adipate (A) or sebacate (Se)) and one aromatic (i.e., the diacid terephthalate (T)) monomer units, their biodegradability (as well as key material properties) can be adjusted by varying the ratio of the respective monomers. A first possibility, as documented for PBAT, is to alter the aromatic diacid T to aliphatic diacid A ratio: increasing the T-to-A ratio decreases PBAT enzymatic hydrolyzability,^17–20^ as well as PBAT soil biodegradability, as determined indirectly by following the gravimetric weight loss of incubated polyester specimens over time.^21–23^ Consistently, enzymatic hydrolysis of PBAT was also found to result in an increase in the T content of the residual (nonhydrolyzed) PBAT.^24^ The decrease in enzymatic hydrolyzability and biodegradability rates with increasing T content has been ascribed to a relative increase in T-enriched crystallites in PBAT, which undergo slower enzymatic hydrolysis as compared to A-enriched amorphous domains of the polyesters.^25^ Furthermore, transitional increases in bulk crystallinities and crystallite sizes of PBAT have been observed during biodegradation.^26,27^ A second possibility to alter the biodegradability of polyesters is to alter the aliphatic diacid, for instance by using Se instead of A. While the biodegradability of polyesters depends on the chemical nature of the monomeric building blocks and not on their feedstock origin,^22^ it is noteworthy that Se is also available from biobased feedstock.^28^ The resulting partially biobased character of PBSeT is attractive in terms of reducing dependence on fossil feedstocks in a circular polymer economy.^29^

While the effects of aliphatic-aromatic polyester chemical structure on biodegradability have long been recognized,^30,31^ studies that determine chemical structure-soil biodegradability relationships for a larger set of these polyesters that systematically differ in their monomeric composition remain forthcoming. For such studies, it is desirable not only to selectively follow polyester mineralization to CO_2_ over time but also to independently verify closed mass balances on initially added polyester carbon over the course of the soil incubations and to quantify potentially present residual biodegradable polyesters at the end of the soil incubations. These two additional measurements allow one to also estimate the amount of polyester carbon incorporated into microbial biomass as the difference between the total nonmineralized carbon and the amount of residual polyest carbon. Closing mass balances on polyester carbon is, however, difficult when using nonlabeled polyesters, as polyester carbon incorporated into biomass cannot be delineated from that of background soil organic matter.

We recently demonstrated the utility of ^13^C-labeled polyesters (with the exemplary polybutylene succinate (PBS)) for quantifying the fraction of polyester that was mineralized to ^13^CO_2_ over the course of the soil incubations using online cavity ring-down spectroscopic (CRDS) detection and to close mass balances on the amount of nonmineralized ^13^C-label that remained in the soil matrix, either as residual polyester or incorporated into biomass/soil organic matter.^32^ Total nonmineralized polyester-added ^13^C was quantified by combusting small soil aliquots in an elemental analyzer coupled to isotope-ratio mass spectrometry (EA-IRMS), while solvent extraction of the soil at the end of the incubation coupled to quantitative ^1^H-nuclear magnetic resonance (NMR) spectroscopy allowed us to quantify the residual PBS remaining in the soil.^33,34^ When combined, these approaches provide detailed information on polyester biodegradation rates and extents and insights into how polyester carbon is microbially utilized in soil.

The objective of the present study was to assess the effect of polyester chemical structure on soil biodegradability for a set of aromatic–aliphatic polyesters that systematically varied in their chemical composition. To this end, we conducted soil incubations with ^13^C-labeled variants of PBAT and PBSeT, both at the same molar ratio of T to total diacids of 50% (i.e., T/(T+A) and T/(T+Se))—to assess the effect of the aliphatic diacid chemistry on biodegradability—and a set of butanediol-^13^C_4_ variants of PBAT that differed in their molar T to total diacid contents of 47%, 30%, 20%, or 0% (i.e., the aliphatic polyester PBA)—to assess the effect of T content on polyester soil biodegradability. For the PBAT and PBSeT comparison, we tested a total of five labeled variants (i.e., three PBAT variants ^13^C-labeled either in the monomers B, T, or A, and two PBSeT variants ^13^C-labeled in the monomers B and T; a variant labeled in Se was not included as the respective labeled monomer was not commercially available) to also assess potential differences in the biodegradation dynamics of the individual monomeric building blocks in these polyesters. The polymerization was restricted to small-scale laboratory reactors (due to the costs of ^13^C-labeled monomers) and resulted in polyester molecular weights lower than those typically achieved by synthesis at the industrial scale and applied in commercial products, such as certified soil-biodegradable mulch films. At the same time, the molecular weight characteristics were consistent between the polyester variants tested herein, thereby allowing the assessment of the effect of polyester monomer composition on soil biodegradability. All polyesters were incubated in an agricultural soil for up to 425 days. During soil incubation, we continuously monitored the mineralization of polyester-^13^C to ^13^CO_2_, followed by quantifying the nonmineralized polyester-added ^13^C, as well as solvent-extracting and quantifying residual polyesters in the soil. We additionally performed incubations of ^13^C-labeled cellulose as a positive biodegradation control in the same soil. Cellulose incubations were conducted at three different added masses (one lower, one matching, and one higher amount than that of the polyesters added) to test for potential decreases in biodegradation rates with increasing mass loadings, which could indicate nutrient limitations during biodegradation (e.g., insufficient nitrogen and phosphorus supply to cellulose-biodegrading soil microorganisms).

## Materials and Methods

### Materials

#### Chemicals

HPLC-grade chloroform (CHCl_3_) and methanol (MeOH) were purchased from Fischer. Deuterated chloroform (CDCl_3_; 99.8% atom D) was from Amar, and 1,4-dimethoxybenzene (DMB; >99%) was from TCI. Butane-1,4-diol (B), ^13^C_4_-butane-1,4-diol, adipic acid (A) (99 atom % ^13^C), 1,6-^13^C_2_-adipic acid, sebacic acid (Se) (99 atom % ^13^C), terephthalic acid (T), and 1-^13^C_1_-terephthalic acid (99 atom % ^13^C) were from Sigma. CHCl_3_ and CDCl_3_ were stored with 4 Å molecular sieves to remove water. All other chemicals were used as received.

#### Polyesters

The ^13^C-labeled PBA_100‑*X*_T_*X*_ and PBSe_50_T_50_ variants were synthesized from the respective monomers according to established polycondensation protocols.^35^ Herein, *X* refers to the percent contribution of T to the total diacid monomeric units in the polyester (Figure 1). The first set of polyester variants were ^13^C-labeled either in B (i.e., P­(^13^C_4_-B)­A_50_T_50_ and P­(^13^C_4_-B)­Se_50_T_50_), in T (i.e., PBA_50_(1-^13^C_1_-T)_50_ and PBSe_50_(1-^13^C_1_-T)_50_), or in A (i.e., PB­(1,6-^13^C_2_-A)_50_T_50_). In the second PBAT series, which varied in T content (and thus *X*), all variants were ^13^C-labeled in B (i.e., P­(^13^C_4_-B)­A_100_T_0_, P­(^13^C_4_-B)­A_80_T_20_), P­(^13^C_4_-B)­A_70_T_30_, and P­(^13^C_4_-B)­A_53_T_47_). Based on the fraction of carbon in the respective monomeric unit selected for labeling to the total carbon in the polyester and the abundance of ^13^C to total C in the labeled monomer used in the synthesis, the ratio of ^13^C-labeled to nonlabeled monomers was adjusted in the synthesis to ensure that the overall ^13^C content in all polyesters was comparable and in the range of 3.5–4.0 atom %. These values and other key physicochemical properties of the polyesters are provided in Section S1. Consistent conditions during the small-scale synthesis of the polyester variants resulted in similar molecular weight characteristics among the variants. We note, however, that during the syntheses, increases in the viscosity of the polyester melts ultimately caused the magnetic stirring in the small reactors to stop, resulting in the polyesters having lower average molecular weights and broader molecular weight distributions than polyesters synthesized at industrial scale and commonly used in commercial products such as certified soil-biodegradable mulch films. Following synthesis, each polyester variant was cryomilled to a powder, which was sieved to collect the fraction with particle diameters between 100 and 300 μm for soil incubations.

**Figure 1:**
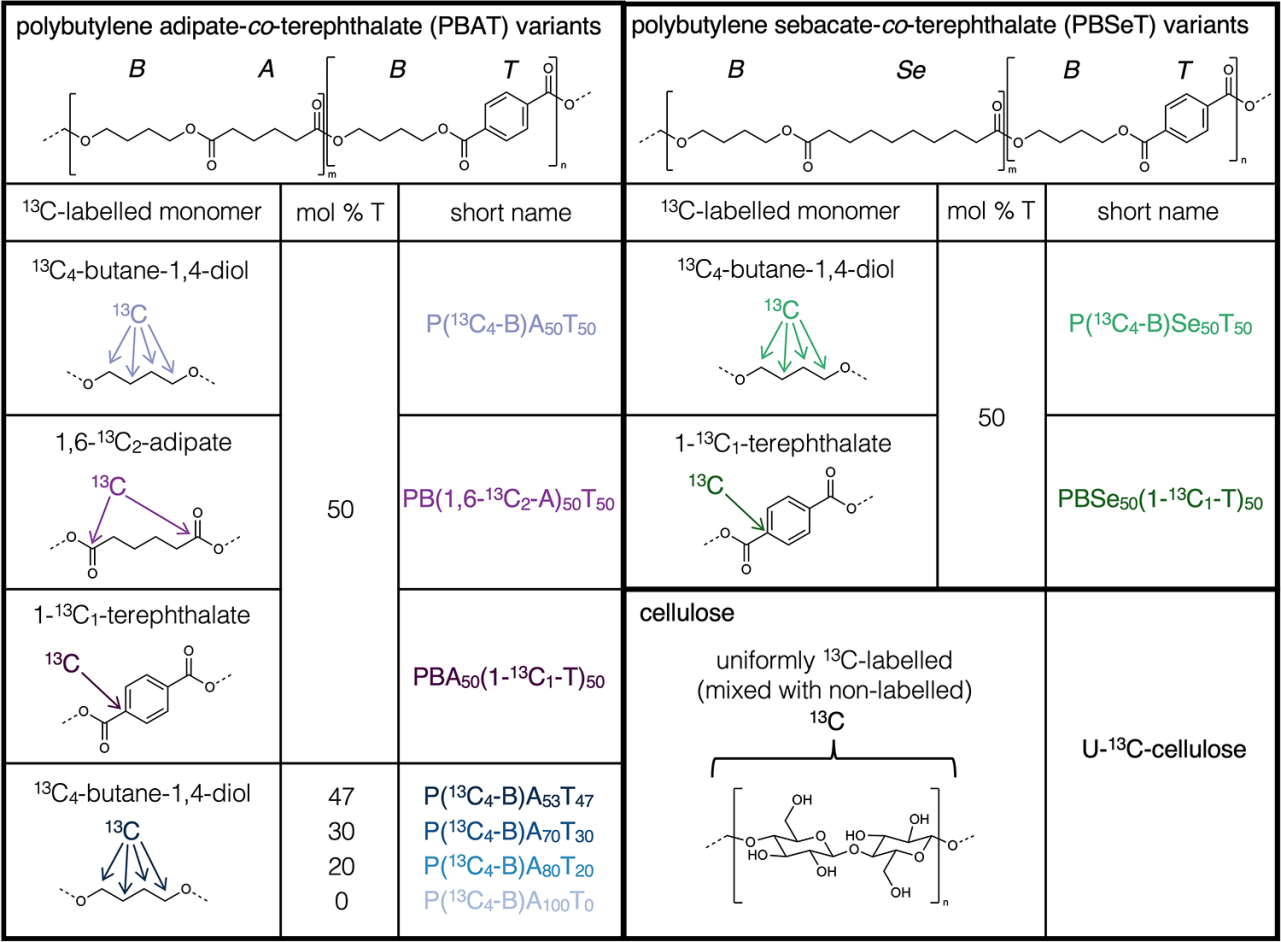
Chemical structures of ^13^C-labeled polyesters and cellulose used in soil incubations. Seven variants of monomer-specific ^13^C-labeled polybutylene adipate-*co*-terephthalate (PBAT) and two variants of polybutylene sebacate-*co*-terephthalate (PBSeT) were used, as shown. Uniformly and fully (≥97%) ^13^C-labeled celluloses were mixed with varying amounts of nonlabeled cellulose (see Materials and Methods text for details).

#### Cellulose

Nonlabeled cellulose (Sigma-Aldrich) and uniformly ^13^C-labeled cellulose (U-^13^C-cellulose; labeling extent of ≥97%; IsoLife, Netherlands) were added at defined ratios to Eppendorf tubes, followed by homogenization of the cellulose mixtures using bead beating with zirconia beads.

#### Soil

The soil for incubations was collected from the Agricultural Center Limburgerhof (Germany) and was used as previously described^32^ (i.e., the soil was air-dried, sieved to 2 mm, stored at 4 °C, and, prior to use in the incubations, the water content was adjusted to 45% of its maximum water-holding capacity (WHC_max_ = 37.3 g H_2_O 100 g^–1^ dry soil) with Milli-Q water (resistivity ≥ 18.2 MΩ cm, TOC ≤ 5 ppb)). The soil was a sandy clay loam according to the USDA soil texture classification system (i.e., particle size distribution by mass of 54.9% in the sand (diameters 50–2 mm), 12.3% in the silt (i.e., 2–50 μm), and 30.8% in the clay fraction (<2 μm)). The organic carbon and total nitrogen contents were 1.14% and 0.11% by weight, respectively.

### Measurement of Polyester- and Cellulose-Derived ^13^CO_2_, ^13^C_mineralized_

Polyester and cellulose mineralization to ^13^CO_2_ was quantified using an automated soil incubation system described previously^32^ with up to 36 incubation bottles (250 mL), each holding 100 g of soil (dry weight equivalent) at 25.0 ± 0.2 °C. The headspace gas in each incubation bottle was continuously exchanged during respirometric analysis at a volumetric flow rate of 24 mL min^–1^. For equilibration, bottles containing soils were run on the system for at least 7 days prior to polyester or cellulose additions. For polyester and cellulose addition, we mixed either ^13^C-labeled polyesters (100 mg per bottle; resulting in an initial polyester concentration of 1 mg polyester g^–1^ dry weight soil) or varying amounts of cellulose, each containing 10 mg of U-^13^C-cellulose (i.e., either only 10 mg ^13^C-cellulose or 10 mg ^13^C-cellulose with either 90 or 490 mg of nonlabeled cellulose; resulting in total added cellulose masses of 10, 100, and 500 mg, respectively). The polyester and cellulose particles were added stepwise to the top of the soil in the incubation bottle, followed by carefully mixing in the particles with a metal spatula to achieve an even distribution in the soil. We note that the experimental data for 100 mg cellulose has already been published in a previous study.^32^

Triplicates of monomer-specific ^13^C-labeled PBAT and PBSeT were incubated in soils for 319 days, at which time we removed one replicate per variant for analyses of nonmineralized polyester carbon, while the remaining duplicate bottles were incubated up to 425 days. Similarly, cellulose incubations were run in triplicate for 139 days, when one bottle was removed, and incubation of the remaining two bottles continued up to 254 days. Incubations of PBAT variants with different T contents were run in triplicate for 145 days. The termination times for the incubations described above were chosen by balancing the amount of data collected vs freeing capacity on the incubation systems. In all cases, the formation of polyester-derived ^13^CO_2_ was still observed when stopping the incubations. The water content of the bottles was monitored gravimetrically and was periodically readjusted using double-deionized water, thereby ensuring that water contents were maintained between 42 and 45% of WHC_max_.

The efflux gas from one incubation bottle at a time was directed to an isotope-specific cavity ring-down spectroscopy analyzer (CRDS; Picarro model G2201-*i*) for the quantification of formed ^13^CO_2_ and ^12^CO_2_. Incubation bottles were measured more frequently in the beginning of the incubations to capture the faster initial mineralization rates. Afterward, bottles were periodically analyzed but stored closed at 25 °C in the dark. We periodically also ran three synthetic air standards containing known concentrations of ^12^CO_2_ and ^13^CO_2_ (Pangas; [CO_2_]_total_ = 400, 500, 700 ppm (±1% relative to concentration) and δ^13^CO_2_ = −5.27, −10.76, −10.21 ‰ (respectively, as quantified by gas-chromatography isotope-ratio mass spectrometry (GC-IRMS)) to the CRDS to correct for minor instrument drift. Each measurement period of a given incubation bottle was 10 min, with a measurement frequency of about 1 s^–1^. Data from the final 3 min in this interval were time-averaged to obtain ^12^CO_2_ and ^13^CO_2_ concentrations per measurement period. Calculations used to determine polyester and cellulose mineralization rates and extents are given in Section S2. The mineralization extents, ^13^C_mineralized_, are expressed as polyester- and cellulose-derived ^13^CO_2_ formed as a percent of polyester- and cellulose-^13^C added to the soil at the onset of the incubation.

### Measurement of Total Nonmineralized Polyester- and Cellulose-Added ^13^C Remaining in Soils, ^13^C_nonmineralized_

After the incubations were terminated, all bottles were transferred to a −20 °C freezer, followed by lyophilization at 0.01 mbar for at least 24 h. The dried soils were passed through a 2 mm sieve, followed by milling approximately half of each dried soil in a vibratory disk mill (RS1, Retsch). To finely disperse residual polyester in soil prior to EA-IRMS analysis, we treated a 5 g subsample of each milled soil with chloroform-sonication.^32^ A polyester-free control soil was treated identically. Details are provided in Section S3-1, including chloroform removal. The chloroform-sonication step was omitted for the soils with cellulose, as it is not soluble in chloroform. A 10 mg subaliquot of each milled and solvent-treated soil for polyester-containing and polyester-free incubations and of the milled soils for cellulose was then weighed into a tin capsule for EA-IRMS analysis.

The tin capsules containing samples were placed on an elemental analyzer (EA) (Thermo Fisher FlashEA 1112) coupled to a continuous flow interface (Thermo Fisher Conflo IV) and an isotope-ratio mass spectrometer (IRMS) (Thermo Fisher Delta V Plus). The EA-IRMS was operated and calibrated as described previously.^32^ We calculated the carbon contents (%C) and δ^13^C values of the combusted soil samples using calibration curves constructed from measurements of various organic compounds with known carbon contents and δ^13^C values, as well as of ^13^C-enriched glucose standards (details in Section S3-2). We converted the determined sample δ^13^C values to ^13^C atom% (see Section S1). The nonmineralized polyester- and cellulose-added ^13^C amounts, ^13^C_nonmineralized_, were calculated as detailed in Section S3 and are expressed as a percent of polyester- or cellulose-^13^C added to the soil at the onset of the incubation.

### Measurement of Residual Polyester in Soils, ^13^C_polymer residual_

Analyses followed extraction procedures previously published.^33,34^ In brief, 3 g of dried and sieved soil from each polyester incubation were transferred into cellulose thimbles, which were placed in Soxhlet extractors (ChemGlass; body volume = 6 mL). The soils were extracted with a 90:10 vol % chloroform-methanol (CHCl_3_:MeOH; total of 10 mL) solvent mixture for 8 h. The solvent was subsequently removed under a stream of compressed air, followed by reconstitution of the dried extract in 2 mL of CDCl_3_ containing a known amount of DMB as an internal standard. The samples were analyzed on a Bruker Avance III 400 MHz NMR spectrometer equipped with a 5 mm BBFO Z-Gradient probe, with settings specified in Section S4. We calculated the extracted polyester mass in each sample as detailed in Section S4, accounting, if occurring, for changes in its chemical composition (i.e., a higher ratio of T to total diacid than for the initially added polyester). We multiplied the extracted polyester mass by the mass ratio of soil in the incubation to the soil extracted and then expressed the residual polyester mass in the soil as a percent of the initially added polyester mass, ^13^C_polymer residual_. Exemplary ^1^H NMR spectra of different polyester chemical structures and DMB, along with details and results of spike-recovery experiments to validate the extraction procedure, are given in Section S4.

## Results and Discussion

### Mineralization of ^13^C-Labeled Polyesters and Cellulose in Soil

#### PBA_50_T_50_ and PBSe_50_T_50_

Figure 2 shows ^13^C-mineralization rates (panels a, c) and corresponding calculated cumulative mineralization extents (panels b, d) for the three monomer-specific ^13^C-labeled PBA_50_T_50_ (panels a, b) and the two monomer-specific ^13^C-labeled PBSe_50_T_50_ variants (panels c, d). Within a few hours of being added to the soil, the PBA_50_T_50_ and PBSe_50_T_50_ variants were mineralized to ^13^CO_2_ (Figure 2a, c; insets). The three variants labeled in B and A (i.e., P­(^13^C_4_-B)­A_50_T_50_, P­(^13^C_4_-B)­Se_50_T_50_, and PB­(1,6-^13^C_2_-A)_50_T_50_) exhibited first maxima in ^13^C-mineralization rates at around six h of incubation. The initial rate maxima of the two variants labeled in T (i.e., PBA_50_(1-^13^C_1_-T)_50_ and PBSe_50_(1-^13^C_1_-T)_50_) were slightly delayed to 18 h of incubation. Following these initial maxima, mineralization rates of all variants decreased to low values after approximately 24 h. At this time, ^13^C_mineralized_ amounted to only small values of 0.5 to 1.25% of the initially added polyester-^13^C (Figure 2b, d; insets). We ascribe these initial mineralization maxima to the microbial utilization of residual labeled monomers and short (possibly cyclic) labeled oligomers that readily diffused out of the bulk at the onset of the incubation and that were sufficiently small to not require enzymatic hydrolysis before being taken up and metabolized by microbes.^32^ The presence of such monomers and oligomers at low mass percentages in polyesters synthesized through polycondensation is well established.^36^ This explanation is consistent with the differences in the occurrence of the initial rate maxima, as these matched the differences in mineralization dynamics of the labeled monomers (i.e., ^13^C_4_-B, 1,6-^13^C_2_-A and 1-^13^C_1_-T) when added directly to soil, as previously reported.^37^

**Figure 2:**
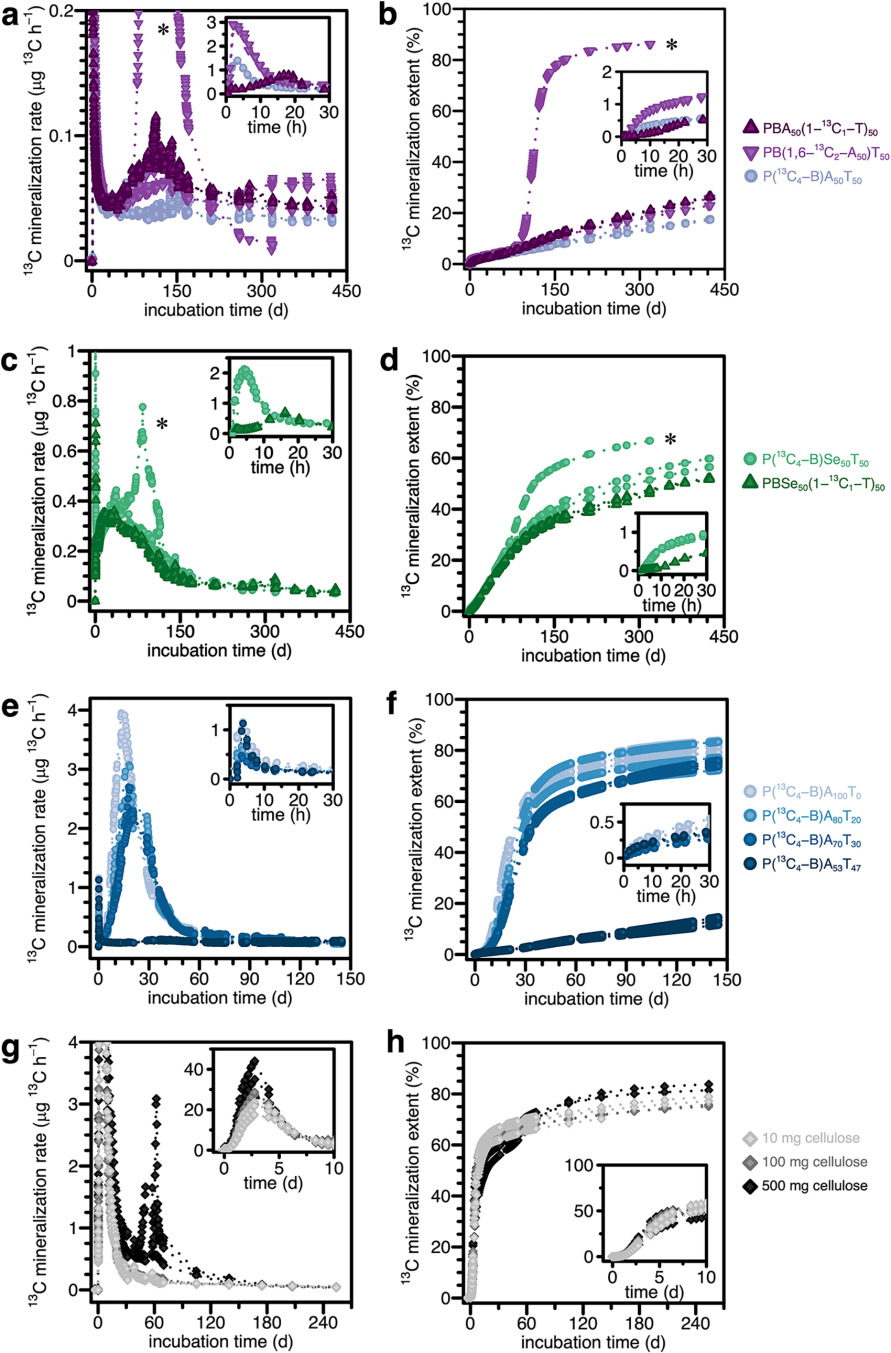
Mineralization rates and cumulative extents of ^13^C-labeled polybutylene adipate-*co*-terephthalate (PBAT) and polybutylene sebacate-*co*-terephthalate (PBSeT) variants and of ^13^C-labeled cellulose in soil. Panels (a, b): monomer-specific ^13^C-labeled PBA_50_T_50_ variants. Panels (c,d) monomer-specific ^13^C-labeled PBSe_50_T_50_ variants. Panels (e, f): butanediol-^13^C-labeled PBA_100‑*X*_T_*X*_ variants with varying molar terephthalate (T) to total diacid contents (i.e., *X* %). All polyester incubations contained 100 mg of the respective variant in 100 g of soil (dry weight equivalents). Panels (g, h): ^13^C-labeled cellulose added in different total amounts of 10, 100, and 500 mg, by mixing 10 mg labeled cellulose with different amounts of nonlabeled cellulose, each in 100 g of soil. Panels b, d, f, and h show cumulative polymer-^13^C mineralization extents (calculated by integration of mineralization rates in panels a, c, e, and g, respectively) expressed as mass percent of polyester- or cellulose-^13^C initially added to soil. Samples marked with an asterisk (*) correspond to those with higher mineralization extents compared with the other replicates of the same polyester variant. For panels (a–d): triplicate soil incubations were run for each polyester variant up to 319 days, when one incubation bottle per variant was terminated, whereas the incubation of the remaining duplicate bottles was continued up to 425 days. For panels (e, f): triplicate incubations were run for each variant for 145 days. For panels (g, h): triplicate incubations were run for each cellulose loading up to 139 days, when one incubation bottle at each mass loading was terminated, and the incubation of the remaining duplicate bottles at each mass loading was continued up to 254 days. Measured points are plotted for each individual replicate, and dotted lines represent linear interpolations between measurement points for those timeframes when bottles continued to be incubated under the same conditions but were not connected to the ^13^CO_2_ detection unit. The cellulose mineralization data at 100 mg has been replotted here from an earlier publication^32^ for comparison.

Following this initial mineralization phase, mineralization rates slowly reincreased to second maxima of around 0.05 to 0.11 μg ^13^C h^–1^ between 100 and 150 days for PBA_50_T_50_ variants and 0.35 to 0.38 μg ^13^C h^–1^ between 15 and 40 days for the PBSe_50_T_50_ variants. Following these maxima, polyester mineralization rates decreased in all incubations to low but above-background values determined from control bottles with only soil until the incubations were terminated. Integration of the ^13^C-mineralization rates of duplicates incubated up to 425 days yielded cumulative mineralization extents of ^13^C_mineralized_ = 26 (±1)%, 25 (±2)%, and 18 (±1)% for PBA_50_(1-^13^C_1_-T)_50_, PB­(1,6-^13^C_2_-A)_50_T_50_, and P­(^13^C_4_-B)­A_50_T_50_, respectively, and 58 (±2)% and 52 (±1)% for P­(^13^C_4_-B)­Se_50_T_50_ and PBSe_50_(1-^13^C_1_-T)_50_, respectively (*n* = 2 per polyester variant, average ± range of two bottles each; note that one bottle of each set of triplicate bottles was already removed after 319 days for quantifying the residual polyester amount and determining the ^13^C mass balance at this intermediate incubation time). This continuous mineralization up to 425 days with a second rate maxima between 100 and 150 days corresponded to the biodegradation of the bulk polyester.

One incubation of each of the above polyester variants was terminated early, after 319 days, for intermediate mass balance assessment and quantification of residual polyester mass (see below). Among these was one incubation bottle of PB­(1,6-^13^C_2_-A)_50_T_50_ and one of P­(^13^C_4_-B)­Se_50_T_50_ with “sudden” enhanced mineralization rates at about 80 days of incubation (marked with asterisks (*) in Figure 2a–d). These two bottles exhibited higher final mineralization extents of ^13^C_mineralized_ = 86% and 67%, respectively, compared to the other two bottles of the same variant (Figure 2b,d). Potential causation for this enhanced biodegradation will be discussed below.

#### PBA_100‑*X*_T_*X*_ with Varying T Content

The mineralization rates and extents of the ^13^C-B-labeled PBA_100‑*X*_T_*X*_ variants with varying T content are shown in Figure 2e,f. The variant with the highest tested T content, PBA_53_T_47_, showed the lowest mineralization rates and final extents (i.e., 13 (±1) % (*n* = 3) after 145 days of incubation; a zoom-in on the data better showing low mineralization rates (≤0.3 μg ^13^C h^–1^) is provided in Section S5). The mineralization rate and extent of the PBA_53_T_47_ variant were higher than those of PBA_50_T_50_ discussed above (i.e., 7 (±1) % after 145 days (*n* = 3); Figure 2b). This finding suggests that PBAT soil biodegradability is highly dependent on the T content at around 50%. Consistently, the PBA_100‑*X*_T_*X*_ variants with lower T contents showed higher mineralization rates and extents—which increased with decreasing T content—after 145 days of incubation: 75 (±1) %, 78 (±6) %, and 82 (±2) % for PBA_70_T_30_, PBA_80_T_20_, and PBA_100_T_0_, respectively (*n* = 3 per polyester variant). While decreases in the T contents from 30 to 0% thus increased soil biodegradability, the comparatively large difference in mineralization between the PBA_70_T_30_ and PBA_53_T_47_ variants supports that the soil biodegradability of PBAT is strongly dependent on T contents between 30 and 47%. As previously reported in the literature, in this T content range, the crystalline domains are formed from B-T units, while for PBAT with lower T contents, these domains are formed from B-A segments.^25^

#### Cellulose

Cellulose mineralization rates increased continuously from the onset of the incubations up to approximately 2 to 3 days, when rates reached maxima of 21 (±3), 26 (±3), and 35 (±8) μg ^13^C h^–1^ in bottles with 10, 100, and 500 mg cellulose, respectively (*n* = 3 per cellulose amount; see Figure 2g, inset). This increase in maximum ^13^C-mineralization rates scaled linearly with the total cellulose ^13^C added (i.e., 10 mg of U-^13^C-labeled cellulose plus approximately 1.1% natural abundance of ^13^C in the 90 and 490 mg of nonlabeled cellulose added) (see Section S6), suggesting similar mineralization dynamics of the labeled and nonlabeled celluloses. Following the initial maxima, the mineralization rates substantially decreased over the next 4 weeks to relatively low values. The secondary, smaller peaks in mineralization rates in the bottles with 500 mg of cellulose after about 50 days of incubation coincided with the addition of water to these soils to maintain a stable soil water content throughout the incubation. The data suggest that the sensitivity of cellulose mineralization rates to soil water content was higher with increasing amounts of cellulose in the soil.

The cumulative mineralization extents showed biphasic behaviors comparable to those for each cellulose treatment (Figure 2h). The first phase corresponded to mineralization of approximately 60% of the added cellulose-^13^C over 10 days, and the second phase was characterized by a flattened slope in the mineralization extent curves. After 254 days, the final mineralization extents were ^13^C_mineralized_ = 78 (±1) %, 75 (±1) %, and 82 (±1) % for incubations with 10, 100, and 500 mg of cellulose added, respectively (*n* = 2, average ± range of two bottles each; note that one bottle of each set of triplicate bottles was already removed after 139 days for determining the cellulose ^13^C mass balance at an intermediate incubation time). Biphasic mineralization of cellulose in soil is consistent with previous reports.^38,39^

We ascribe the initial phase with high mineralization rates to fast and extensive microbial utilization of cellulose both for energy generation (respiratory production of CO_2_) and for biomass buildup. The onset of the second phase then occurred following consumption of most of the added cellulose and was due to slower turnover of microbial bio­(necro)­mass containing cellulose-derived ^13^C. In this case, soil microorganisms had comparable metabolic utilization patterns of the cellulose at the three mass loadings, with approximately 60% of the cellulose carbon being mineralized to CO_2_ and 40% of the carbon being incorporated. This carbon use efficiency of approximately 40% is fully consistent with previous studies and theoretical considerations, although not experimentally proven in our experiments due to solvent extraction and thus subsequent quantification of residual cellulose being impossible.^40–42^ More importantly, comparable mineralization dynamics at the three different cellulose mass loadings strongly suggest that substrate concentrations used in our incubations were sufficiently low to ensure that microbial biodegradation of cellulose and polyesters was not limited by the supply of nitrogen or phosphorus to the cells (which would have slowed biodegradation at high mass loadings). The possibility to run incubations at such low polyester and cellulose concentrations (due to high signal-to-noise when quantifying ^13^C) is a unique advantage of using ^13^C-isotopically labeled instead of nonlabeled polymers.

### Closing Mass Balances on Polyester- and Cellulose-Added ^13^C over the Course of the Incubations

Figure 3 (left side) shows the total amount of nonmineralized polyester- and cellulose-added ^13^C remaining in the soil at the end of the incubations (^13^C_nonmineralized;_ expressed in % of polyester- or cellulose-^13^C initially added to the soils), as quantified by EA-IRMS on small soil aliquots. Figure 3 (right side) also shows the corresponding final mineralization extents (^13^C_mineralized_) replotted from Figure 2. Exact ^13^C_nonmineralized_ and ^13^C_mineralized_ values are also provided in Section S7.

**Figure 3:**
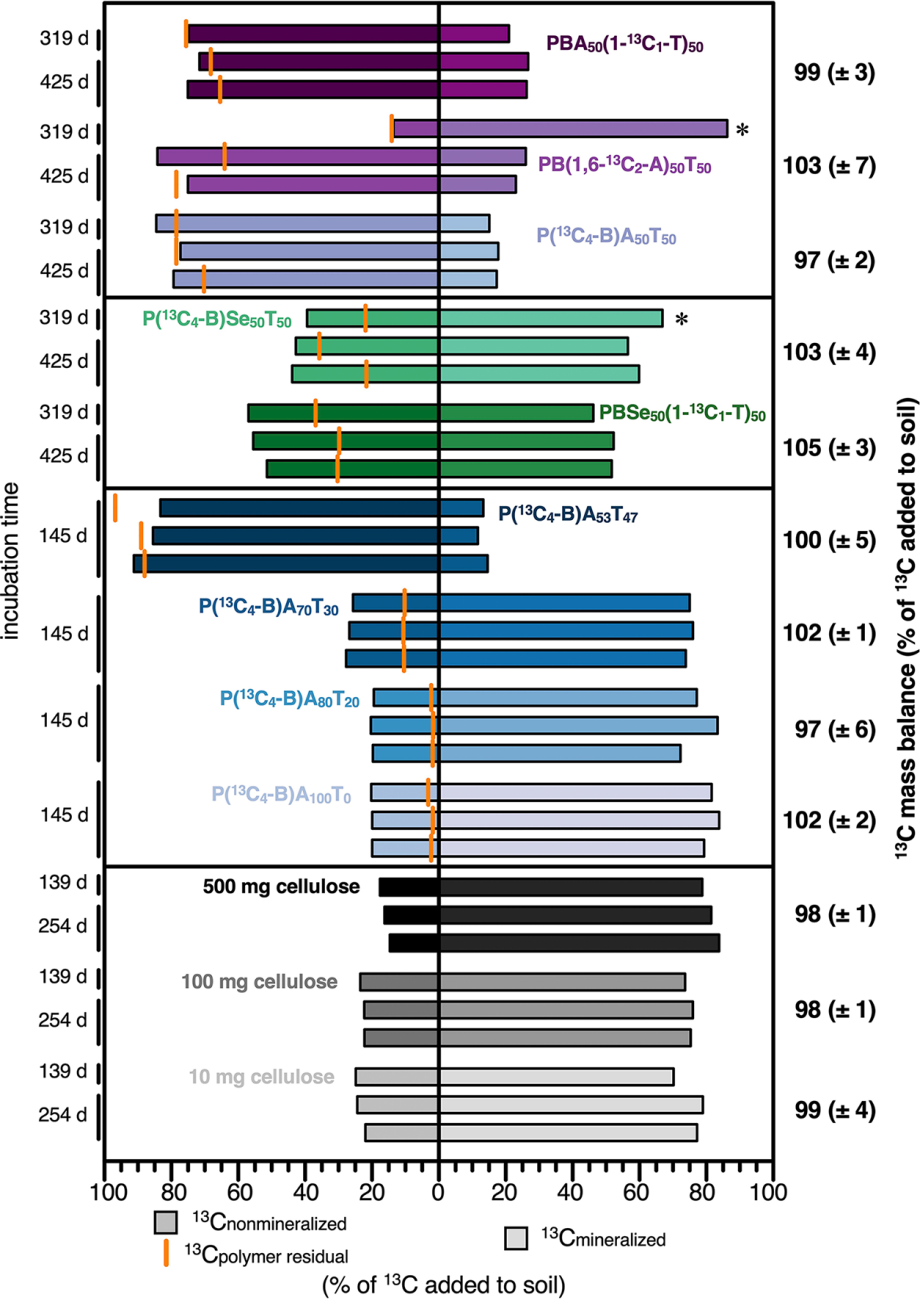
Quantification and characterization of nonmineralized polymer-added ^13^C remaining in soils after incubation. Bars on the left represent polyester- and cellulose-added ^13^C that did not mineralize over the course of the incubation and thus remained in the soils, ^13^C_nonmineralized_ (expressed as a percent of polyester- and cellulose-added ^13^C initially added to the soil). Bars on the right represent cumulative final amounts of polyester-^13^C mineralized (^13^C_mineralized_; expressed as a percent of polyester- and cellulose-added ^13^C initially added to the soil) in each bottle at the end of the incubations (respective incubation times are indicated to the left of the box; this data corresponds to the final mineralized amounts of polyesters and cellulose shown in Figure 2; samples marked with an asterisk (*) correspond to those with higher mineralization extents compared to the other replicates of the same polyester variant). The mass balance on both polyester- and cellulose-added ^13^C was closed, as indicated by the good agreement between the sum of ^13^C_nonmineralized_ and ^13^C_mineralized_ with the initially added amounts of ^13^C (mass balances in percent of polyester- and cellulose-added ^13^C initially added to the soil to the right of the plot (average ± standard deviation of triplicate incubation bottles)). The orange vertical lines plotted on the left side for each incubation bottle represent the quantified amounts of polyester carbon that remained in the soil, ^13^C_polymer residual_, as determined by Soxhlet extraction of soil aliquots followed by ^1^H NMR spectroscopy analysis (also expressed as a mass % of added polyester carbon, converted to polyester-^13^C added to the soil at the onset of the incubation). The data for 100 mg of cellulose has been reported previously.^32^

As expected, ^13^C_nonmineralized_ of both the polyester- and cellulose-added ^13^C in the soil decreased with increasing mineralization extents for the respective incubation. More importantly, the sum of ^13^C_nonmineralized_ and ^13^C_mineralized_ corresponded to, on average, 97–105% of the initially added ^13^C (averages of triplicate incubations per polyester and cellulose variant). This finding implies closed mass balances on polyester- and cellulose-added ^13^C over the course of the incubations, including for the two incubation bottles of PB­(1,6-^13^C_2_-A)­T and P­(^13^C_4_-B)­SeT with higher mineralization extents (marked with asterisks (*) in Figure 3), thereby confirming that these incubations indeed showed enhanced biodegradation. Accurate quantification and tracking of polymer-added ^13^C and the capability to close mass balances on polymer-^13^C over the course of long-term (soil) incubations is another advantage of using ^13^C-labeled polymers as compared to using nonlabeled polymers. For the latter, closing mass balances are impaired as polymer-added carbon remaining in the soil cannot readily be delineated from carbon in soil organic matter.

### Assessing the Contribution of Residual Polyester to the Nonmineralized Polyester-Added ^13^C

The amounts of residual PBAT and PBSeT, ^13^C_polymer residual_ (expressed as the percent of initially added polyester C extracted, converted to polyester-^13^C added to the soil at the onset of the incubation) that remained in the soils at the end of the incubations are plotted as vertical orange lines on the left side. This assessment could not be conducted for cellulose as it is not solvent-extractable. It is important to note here that incubation experiments were terminated prior to ^13^CO_2_ formation plateauing, implying that PBAT and PBSeT biodegradation was still ongoing. After 425 days of incubation, the three different monomer-specific ^13^C-labeled PBA_50_T_50_ variants had similar ^13^C_polymer residual_ values of 67 (±1) %, 71 (±7) %, and 74 (±4) % for PBA_50_(1-^13^C_1_-T)_50_, PB­(1,6-^13^C_2_-A)_50_T_50_, and P­(^13^C_4_-B)­A_50_T_50_, respectively (average ± range for duplicate samples per polyester variant). By comparison, ^13^C_polymer residual_ after 425 days was lower for the two ^13^C-labeled PBSe_50_T_50_ variants at 29 (±7) % and 30 (±1) % (*n* = 2) for P­(^13^C_4_-B)­Se_50_T_50_ and PBSe_50_(1-^13^C_1_-T)_50_, respectively, consistent with their higher extents of mineralization. Finally, ^13^C_polymer residual_ of the PBA_100‑*X*_T_*X*_ series with varying T content was 3 (±1) %, 2 (±1) %, 10 (±1) %, and 91 (±5) %, (*n* = 3) for PBA_100_T_0_, PBA_80_T_20_, PBA_70_T_30_, and PBA_53_T_47_, respectively. We note that the few cases in which calculated ^13^C_polymer residual_ exceeded ^13^C_nonmineralized_ likely resulted from the 3 g of soil being extracted containing a nonrepresentative, slightly higher amount of polyester than expected for a representative subsample taken from the 100 g of bulk soil. This finding implies that downsizing soil aggregates by sieving the entire soil prior to subsampling 3 g for Soxhlet extraction did not completely remove the heterogeneity in polyester distribution in the soil. This explanation is favored over ^13^C_nonmineralized_ values being inaccurate, given that ^13^C_nonmineralized_ is indirectly validated by closed mass balances when combined with ^13^C_mineralized_. Despite potential small uncertainties caused by extracting soil aliquots, ^13^C_polymer residual_ for all polyester variants decreased with decreasing ^13^C_nonmineralized_, as expected (see Section S8).

### Assessment of Chemical Structure-Soil Biodegradability Relationships of Biodegradable Polyesters

The use of ^13^C-labeled polyester variants differing in their chemical composition and the combined determination of ^13^C_mineralized_, ^13^C_nonmineralized_, and ^13^C_polymer residual_ allows for a detailed analysis of the polyester chemical structure-biodegradability dependencies. The observed differences between variants must result from the polyester chemical structure, as the same soil and incubation conditions were used in all experiments.

#### Variations in the Aliphatic Diacid

Mineralization extents after 425 days were much higher for PBSe_50_T_50_ (i.e., ^13^C_nonmineralized_ from 52 to 59%) than for PBA_50_T_50_ (from 17 to 26%), implying that variations in the aliphatic diacid in aliphatic-aromatic copolyesters strongly affect polyester biodegradation. By subtracting the amounts of extracted residual polyesters, ^13^C_polymer residual_, from the amounts of total polyester-added ^13^C left nonmineralized in the soils, ^13^C_nonmineralized_, we could estimate the amounts of polyester-^13^C that were incorporated into microbial biomass, ^13^C_biomass_ (note, however, that this analysis calls for a careful interpretation, particularly for polyesters with low mineralization extents, due to several confounding factors addressed in Section S9). For PBSe_50_T_50_, we estimated ^13^C_biomass_ to be 19 (±6) % across all incubations (*n* = 6) (Figure 4a), revealing that considering only mineralization extents, despite them being high, would result in substantially underestimating total biodegradation extents of this polyester. While biomass incorporation also seems to have occurred for the PBA_50_T_50_ and PBA_53_T_47_ variants, its extent was much smaller, reflecting the overall lower mineralization extents (Figure 4a) (i.e., the PBA_50_T_50_ and PBA_53_T_47_ variants exhibited low carbon incorporation into biomass of ^13^C_biomass_ 3 (±9) % (*n* = 12)). The high uncertainty in ^13^C_biomass_ results from ^13^C_polymer residual_ in some bottles exceeding ^13^C_nonmineralized_ (see above discussion), resulting in tentatively negative biomass incorporation (see Figure 4a). The low incorporation of ^13^C into biomass for the one PB­(1,6-^13^C_2_-A)_50_T_50_ incubation that exhibited a high mineralization extent (see sample marked with an asterisk (*) in Figure 4a) likely reflects extensive decarboxylation of the ^13^C in the carboxylate carbons of A into ^13^CO_2_, while the four nonlabeled inner carbons of A of this variant were relatively more extensively incorporated into biomass. This pattern of carbon position-specific preferential mineralization or biomass incorporation has been previously demonstrated and discussed in detail for soil incubations of position-specific ^13^C-labeled succinate (S; i.e., 1,4-^13^C_2_-S and 2,3-^13^C_2_-S) and the corresponding position-specific ^13^C-labeled variants of the S-containing polyester polybutylene succinate (PBS).^32^ The monomer-specific labeling of PBA_50_T_50_ and PBSe_50_T_50_ further allowed demonstrating that carbon from all labeled monomeric units (i.e., A, B, and T) biodegraded to similar extents—thereby ruling out that any of these building blocks underwent delayed biodegradation. This finding is consistent with previous work demonstrating rapid biodegradation of ^13^C-labeled B, A, and T when added as monomers to soil, suggesting that low-molecular-weight monomeric and oligomeric polymer breakdown products do not accumulate in soils.^37^ We note that ^13^C-labeled Se was not commercially available at the time of synthesis. Overall, faster and more extensive biodegradation of PBSe_50_T_50_ than PBA_50_T_50_ in the tested soil is consistent with previously reported differences in PBAT and PBSeT degradation in soil and compost assessed indirectly by gravimetric weight loss measurements.^16,21,43^

**Figure 4:**
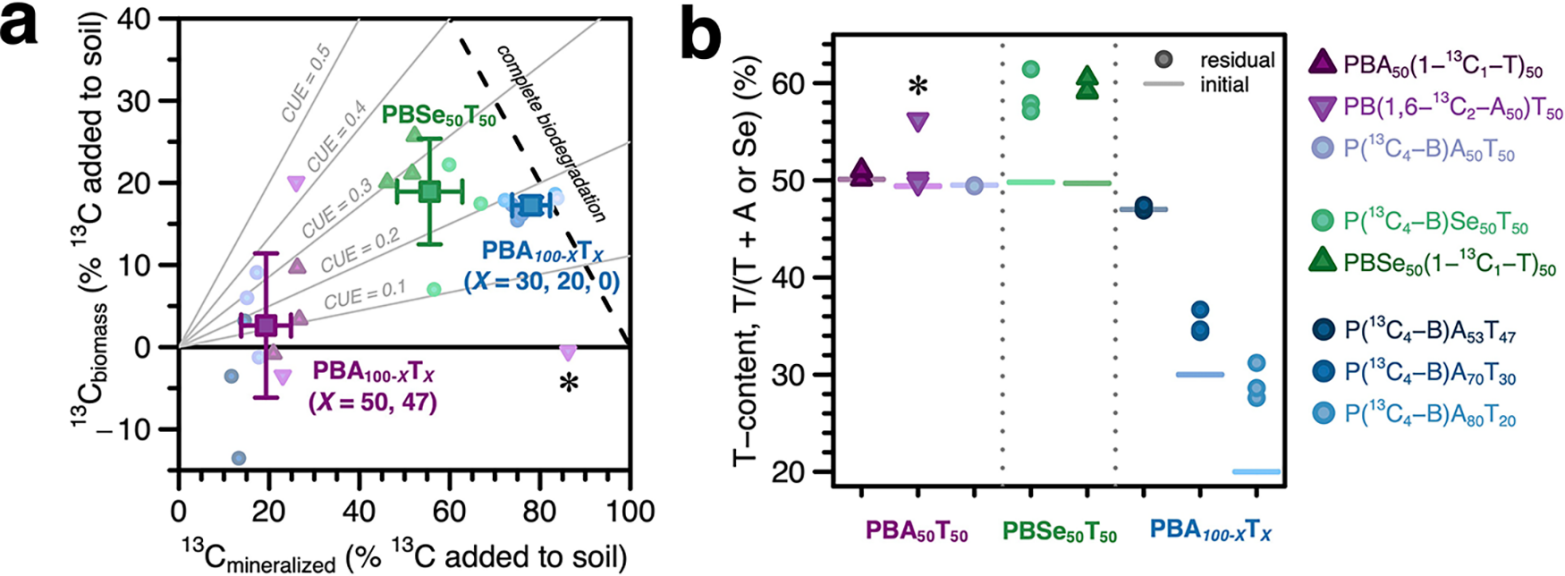
Dependence of polyester biodegradation on chemical structure. (a) Microbial utilization of polyester carbon in soil incubations, shown as the amounts of polyester-^13^C incorporated into microbial biomass (^13^C_biomass_) vs the extents of polyester-^13^C mineralized (^13^C_mineralized_) at the end of incubations (^13^C_mineralized_ values are replotted from Figure 3). Small, faint symbols represent values for individual incubations; large squares and error bars represent averages and standard deviations, respectively, for the indicated polyester variants (note that the inverted triangle marked with an asterisk (*) represents one replicate of the PB­(1,6-^13^C_2_-A)_50_T_50_ variant that mineralized to an exceptionally high extent; therefore, its value was excluded from the average). The dashed black line corresponds to states of complete polyester biodegradation (i.e., ^13^C_mineralized_ + ^13^C_biomass_ = 100%). Light gray lines represent different theoretical values of the carbon use efficiencies (CUE, i.e., ^13^C_biomass_/(^13^C_biomass_+^13^C_mineralized_); value indicated by the respective label in the plot). (b) Terephthalate (T) contents in residual polyesters extracted from soils at the end of incubations (symbols, “residual”; data points correspond to three replicate incubations) relative to those of the starting polyesters (light horizontal lines, “initial”), as determined using proton nuclear magnetic resonance spectroscopy (^1^H NMR; see Materials and Methods for details on measurements and calculations).

While the cause for enhanced mineralization in one of the PB­(1,6-^13^C_2_-A)_50_T_50_ and P­(^13^C_4_-B)­Se_50_T_50_ replicate incubations remains unidentified, two explanations seem plausible. First, these two incubation bottles may have contained a microbial strain that is particularly competent in biodegrading PBAT and PBSeT and began to proliferate and degrade these polyesters after approximately 80 days of incubation. Second, a random adaptation of one (or more) microbial strain in these two bottles may have enhanced their potential to biodegrade the two polyesters. These adaptations may have involved the upregulation of the production and exudation of highly active extracellular esterases,^44^ as enzymatic ester bond hydrolysis is generally thought to control the overall biodegradation rates of PBAT and PBSeT in soil.^45^ Irrespective of which explanation holds true, the finding of sudden enhanced biodegradation highlights that soil microbial communities can become more adept, through either adaptation or selection of specialist microbial strains, and that repeated inputs of biodegradable polyesters to soils could enhance biodegradation rates.

#### Variations in the T Content of PBAT

As compared to the PBA_50_T_50_ and PBA_53_T_47_ variants, the PBA_70_T_30_, PBA_80_T_20_, and PBA_100_T_0_ variants not only underwent much more extensive mineralization but also showed substantial incorporation of polyester carbon into microbial biomass (i.e., ^13^C_biomass_= 17 (±1) % (*n* = 9); Figure 4a). More extensive biomass incorporation is consistent with PBSe_50_T_50_ biodegradation, as this polyester also underwent fast and extensive mineralization in soils. For such polyesters, incubation experiments determining only ^13^C_mineralized_ (*x*-axis in Figure 4a) underestimate true biodegradation by the extent of ^13^C_biomass_ (*y*-axis in Figure 4a; the corresponding carbon use efficiencies (CUEs) are shown as gray lines). In this figure, the “true” extent of biodegradation corresponds to the vertical offset between the data points and the dashed black line representing “complete biodegradation” (i.e., ^13^C_mineralized_ + ^13^C_biomass_ = 100%) (and not the vertical offset between the dashed line and the *x*-axis). The finding of low ^13^C_biomass_ for polyesters which underwent slower mineralization may not necessarily imply that there was no incorporation but that cycling of polyester-derived ^13^C through the microbial biomass pool into ^13^CO_2_ occurred at rates comparable to (or faster than) the rate by which ^13^C was incorporated into microbial biomass. In such cases, the steady-state ^13^C_biomass_ would be small (as shown here for PBAT with T contents ≥ 47%), which aligns with modeled biodegradation dynamics of PBS in soil.^32^

We ascribe the pronounced decrease in soil biodegradability from the PBA_100_T_0_, PBA_80_T_20_, and PBA_70_T_30_ variants to the PBA_53_T_47_ (and PBA_50_T_50_) variant to an underlying decrease in the enzymatic hydrolyzability of these polyesters.^18,20^ Higher T contents lead to increased contents of T-enriched microcrystalline domains, which undergo slower enzymatic hydrolysis. This explanation is directly supported by observed changes in the chemical composition of the extracted residual PBAT and PBSeT at the end of the incubations based on their ^1^H NMR spectra. Both the extracted residual P­(^13^C_4_-B)­A_70_T_30_ and P­(^13^C_4_-B)­A_80_T_20_ variants showed increased T contents of 35 (±1) % and 29 (±2) % (*n* = 3 per variant), respectively, as compared to the T contents of the initially added polyesters of 30 and 20%, respectively. Similarly, the residual PBSeT extracted from the soils also had elevated T contents of 59 (±2) % (*n* = 6) as compared to 50% in the initially added PBSe_50_T_50_. An increased T content of 56% (from initially 50%) was also found for the residual PB­(1,6-^13^C_2_-A)_50_T_50_ in the one incubation bottle that showed enhanced biodegradation (inverted triangle marked with an asterisk (*) in Figure 4b), demonstrating that the T-enrichment also affected higher T content PBAT variants when undergoing extensive biodegradation. Our finding that extensive biodegradation results in increased T contents in the residual polyesters is in agreement with previous reports of relative increases in T contents in aromatic-aliphatic polyesters upon both abiotic hydrolysis^17,46^ as well as incubations with isolated soil microorganisms^24^ or in bulk soils.^37,47,48^ It is important to emphasize that while there is enrichment in T during the biodegradation, the terephthalate units are also subject to mineralization. This is seen in the two variants that were ^13^C-labeled in T (i.e., PBA_50_(1-^13^C_1_-T)_50_ and PBSe_50_(1-^13^C_1_-T)_50_), both of which showed production of ^13^CO_2_, unequivocally demonstrating that biodegradation of PBA_100‑*X*_T_*X*_ and PBSe_100‑*X*_T_*X*_ also involves their terephthalate components.

### Implications

This work established that changes in the monomer composition of aliphatic-aromatic copolyesters can strongly affect their biodegradation rates and extents in soils. Compared to polybutylene adipate-*co*-terephthalate with (close to) equimolar contents of T and A, mineralization rates and biodegradation extents in soil increased largely when replacing the diacid A by Se (i.e., increasing the length of the aliphatic diacid from six to ten carbon atoms) and when decreasing the T content while increasing the A content. These results highlight a unique potential of the modular composition of aliphatic-aromatic polyesters: the possibility of tuning their biodegradation rates through alteration of their chemical composition. Polyester chemical composition may thus present a means to establish a desired polyester biodegradability for not only soils but also other natural (e.g., freshwater and marine) or engineered (e.g., compost) receiving environments. For instance, it is conceivable that a set of commercially certified soil-biodegradable products composed of aliphatic-aromatic polyesters, such as thin mulch films, could be marketed that vary in their monomeric polyester compositions to match the biodegradation potential of specific receiving soils. Clearly, such an approach requires also a more detailed understanding of the factors driving variations in the biodegradation potential of soils^49,50^ (or other receiving systems).^27,51,52^ It is noteworthy, however, that the rate of biodegradation can also be adjusted by other approaches, such as compounding with other polymers or modifying the crystallinity by altering the production process. Furthermore, we note that the average molecular weights of the PBAT variants synthesized in the reported small-scale laboratory reactors were lower than those in PBAT (and other polyesters) commonly used in commercial products (e.g., PBAT used in certified soil-biodegradable mulch films). Considering that polyester biodegradation rates may decrease with increasing molecular weight, biodegradation rates determined herein may be higher than those of polyesters with the same or comparable chemical composition in commercial products.

Solvent extraction of residual polyesters from soils followed by ^1^H NMR analysis is shown to be useful not only for accurately quantifying the true extent of polyester biodegradation (which may be underestimated by mineralization measurements alone) but also for determining changes in the monomeric compositions of a polyester during its biodegradation. The observed relative increase in T contents in the residual polyesters can be rationalized based on the established faster biodegradation of A- and Se-enriched amorphous domains and the slower biodegradation of T-enriched crystalline domains. This finding thus highlights how chemical structure-soil biodegradability assessments can be linked to a molecular-level enzymatic hydrolysis reaction. This finding also supports that the assessment of polyester hydrolyzability by natural extracellular esterases has predictive power for their environmental biodegradability. Our work further supports the use of solvent extraction of residual polyesters coupled to their quantification and chemical characterization in future biodegradation studies. This is especially interesting for studies following polyester (or products composed thereof) biodegradation in the open environment (e.g., field soils) in which mineralization measurements are not feasible (or even impossible).^34^ In such studies, changes in the monomer composition of extracted, residual polyester can be used as an independent indicator (besides decreasing polyester concentrations) that biodegradation has occurred. It remains to be established how such transient changes in the chemical composition of residual polyesters during biodegradation affect their subsequent biodegradation rates. It may be possible to counteract potential slowdown in subsequent biodegradation by other chemical modifications of the polyester structure or by using polymer blends that lead to more rapid or synergistic biodegradation.

Quantification of the residual polyesters, combined with mineralization extents, revealed a more extensive incorporation of polyester carbon into microbial biomass for polyesters undergoing fast biodegradation. Polyesters undergoing fast and extensive mineralization may have substantial polyester carbon assimilated into the microbial biomass. By comparison, for slowly mineralizing polyesters, little of the nonmineralized polyester carbon may be incorporated in microbial biomass. Quantification of C_polymer residual_ does not require ^13^C-labeling and thus is a valuable end point measurement also for standard incubations following polyester mineralization: in combination, quantification of C_mineralized_ and C_polymer residual_ can disclose the true extent of polyester biodegradation and of polyester-C incorporation into microbial biomass.

## Supplementary Material



## Notes

Physicochemical properties of polyesters (Section S1); calculations of polymer mineralization rates and extents (Section S2); details on quantifying total nonmineralized polyester- and cellulose-added ^13^C remaining in soils, ^13^C_nonmineralized_ (Section S3); details on quantifying residual polyester in soils, C_polymer residual_ (Section S4); mineralization dynamics of P­(^13^C_4_-B)­A_100‑*X*_T_*X*_ in soil (Section S5); mineralization rates of cellulose-^13^C vs total cellulose-^13^C added to soil (Section S6); compilation of ^13^C_mineralized_, ^13^C_nonmineralized_, and ^13^C_polymer residual_ data for all incubations (Section S7); correlation between ^13^C_polymer residual_ and ^13^C_nonmineralized_ for polyesters at the end of soil incubations (Section S8); interpretation of ^13^C_biomass_ (Section S9) (PDF)

## References

[1] Geyer R., Jambeck J. R., Law K. L. P. (2017). Use, and Fate of All Plastics Ever
Made. Sci. Adv.

[2] Chae Y., An Y. J. (2018). Current Research
Trends on Plastic Pollution and Ecological Impacts
on the Soil Ecosystem: A Review. Environ. Pollut.

[3] Bläsing M., Amelung W. (2018). Plastics in Soil: Analytical
Methods and Possible Sources. Sci. Total Environ.

[4] Zubris K. A. V., Richards B. K. (2005). Synthetic Fibers
as an Indicator of Land Application
of Sludge. Environ. Pollut.

[5] Changrong Y., Wenqing H., Turner N. C., Enke L., Qin L., Shuang L. (2014). Plastic-Film Mulch
in Chinese Agriculture: Importance
and Problems. World Agric..

[6] Liu E. K., He W. Q., Yan C. R. (2014). “White
Revolution”
to “White Pollution” - Agricultural Plastic Film Mulch
in China. Environ. Res. Lett.

[7] Albertsson A. C., Hakkarainen M. (2017). Designed to
Degrade. Science.

[8] Gross R. A., Kalra B. (2002). Biodegradable Polymers
for the Environment. Science.

[9] Hemphill D. D. (1993). Agricultural
Plastics as Solid Waste: What Are the Options for Disposal?. HortTechnology.

[10] Kasirajan S., Ngouajio M. (2012). Polyethylene and Biodegradable
Mulches for Agricultural
Applications: A Review. Agron. Sustainable Dev.

[11] Brodhagen M., Peyron M., Miles C., Inglis D. A. (2015). Biodegradable Plastic
Agricultural Mulches and Key Features of Microbial Degradation. Appl. Microbiol. Biotechnol.

[12] Wolf C., Wenzel M., Fischer B., Bertling R., Jelen E., Hennecke D., Weinfurtner K., Roß-Nickoll M., Hollert H., Weltmeyer A., Bitter K., Ruiz P., Banduka D., Tuerk J., Blank L. M. (2025). iMulch: An Investigation
of the Influence of Polymers on a Terrestrial Ecosystem Using the
Example of Mulch Films Used in Agriculture. Environ. Sci. Eur.

[13] Lucas N., Bienaime C., Belloy C., Queneudec M., Silvestre F., Nava-Saucedo J. E. (2008). Polymer Biodegradation: Mechanisms
and Estimation Techniques - A Review. Chemosphere.

[14] Kijchavengkul T., Auras R., Rubino M., Ngouajio M., Thomas Fernandez R. (2006). Development
of an Automatic Laboratory-Scale Respirometric System to Measure Polymer
Biodegradability. Polym. Test.

[15] Künkel, A. ; Becker, J. ; Börger, L. ; Hamprecht, J. ; Koltzenburg, S. ; Loos, R. ; Schick, M. B. ; Schlegel, K. ; Sinkel, C. ; Skupin, G. Polymers, Biodegradable In Ullmann’s Encyclopedia of Industrial Chemistry Wiley-VCH Verlag GmbH & Co. KGaA: Weinheim, Germany, 2016pp. 1–29 10.1002/14356007.n21_n01.pub2.

[16] Dietrich, B. ; Siegenthaler, K. O. ; Skupin, G. ; Künkel, A. ; Yamamoto, M. Aliphatic-Aromatic Polyester WO 2,010,034,710 A1 2010.

[17] Herrera R., Franco L., Rodríguez-Galán A., Puiggalí J. (2002). Characterization and Degradation Behavior of Poly­(Butylene
Adipate-Co-Terephthalate)­s. J. Polym. Sci.,
Part A: Polym. Chem.

[18] Marten E., Müller R.-J., Deckwer W.-D. (2005). Studies on the Enzymatic Hydrolysis
of Polyesters. II. Aliphatic–Aromatic Copolyesters. Polym. Degrad. Stab.

[19] Perz V., Baumschlager A., Bleymaier K., Zitzenbacher S., Hromic A., Steinkellner G., Pairitsch A., Łyskowski A., Gruber K., Sinkel C., Küper U., Ribitsch D., Guebitz G. M. (2016). Hydrolysis of Synthetic
Polyesters
by *Clostridium Botulinum* Esterases. Biotechnol. Bioeng.

[20] Zumstein M. T., Rechsteiner D., Roduner N., Perz V., Ribitsch D., Guebitz G. M., Kohler H.-P.-P. E., McNeill K., Sander M. (2017). Enzymatic
Hydrolysis of Polyester Thin Films at the Nanoscale: Effects of Polyester
Structure and Enzyme Active-Site Accessibility. Environ. Sci. Technol.

[21] Witt U., Müller R. J., Deckwer W. D. (1995). New Biodegradable Polyester-Copolymers
from Commodity Chemicals with Favorable Use Properties. J. Environ. Polym. Degrad.

[22] Witt U., Yamamoto M., Seeliger U., Müller R. J., Warzelhan V. (1999). Biodegradable Polymeric Materials—Not
the Origin
but the Chemical Structure Determines Biodegradability. Angew. Chem. Int. Ed.

[23] Xu P.-Y., Liu T.-Y., Huang D., Zhen Z.-C., Lu B., Li X., Zheng W.-Z., Zhang Z.-Y., Wang G.-X., Ji J.-H. (2023). Enhanced
Degradability of Novel PBATCL Copolyester: Study on the Performance
in Different Environment and Exploration of Mechanism. Eur. Polym. J.

[24] Rüthi J., Cerri M., Brunner I., Stierli B., Sander M., Frey B. (2023). Discovery of Plastic-Degrading Microbial Strains Isolated from the
Alpine and Arctic Terrestrial Plastisphere. Front. Microbiol.

[25] Gan Z., Kuwabara K., Yamamoto M., Abe H., Doi Y. (2004). Solid-State
Structures and Thermal Properties of Aliphatic-Aromatic Poly­(Butylene
Adipate-Co-Butylene Terephthalate) Copolyesters. Polym. Degrad. Stab.

[26] Svoboda P., Dvorackova M., Svobodova D. (2019). Influence
of Biodegradation on Crystallization
of Poly (Butylene Adipate-Co-Terephthalate). Polym. Adv. Technol.

[27] Liu B., Guan T., Wu G., Fu Y., Weng Y. (2022). Biodegradation
Behavior of Degradable Mulch with Poly (Butylene Adipate-Co-Terephthalate)
(PBAT) and Poly (Butylene Succinate) (PBS) in Simulation Marine Environment. Polymers.

[28] Cornils, B. ; Lappe, P. Dicarboxylic Acids, Aliphatic. In Ullmann’s Encyclopedia of Industrial Chemistry; John Wiley & Sons, Ltd: 2014, pp. 1–18. 10.1002/14356007.a08_523.pub3.

[29] Haque F. M., Ishibashi J. S. A., Lidston C. A. L., Shao H., Bates F. S., Chang A. B., Coates G. W., Cramer C. J., Dauenhauer P. J., Dichtel W. R., Ellison C. J., Gormong E. A., Hamachi L. S., Hoye T. R., Jin M., Kalow J. A., Kim H. J., Kumar G., LaSalle C. J., Liffland S., Lipinski B. M., Pang Y., Parveen R., Peng X., Popowski Y., Prebihalo E. A., Reddi Y., Reineke T. M., Sheppard D. T., Swartz J. L., Tolman W. B., Vlaisavljevich B., Wissinger J., Xu S., Hillmyer M. A. (2022). Defining the Macromolecules
of Tomorrow through Synergistic Sustainable Polymer Research. Chem. Rev.

[30] Kijchavengkul T., Auras R. (2008). Compostability of Polymers. Polym. Int.

[31] Stevens E. S. (2003). What Makes
Green Plastics Green?. BioCycle.

[32] Nelson T. F., Baumgartner R., Jaggi M., Bernasconi S. M., Battagliarin G., Sinkel C., Künkel A., Kohler H.-P. E., McNeill K., Sander M. (2022). Biodegradation of Poly­(Butylene
Succinate) in Soil Laboratory Incubations Assessed by Stable Carbon
Isotope Labelling. Nat. Commun.

[33] Nelson T. F., Remke S. C., Kohler H. P. E., McNeill K., Sander M. (2020). Quantification
of Synthetic Polyesters from Biodegradable Mulch Films in Soils. Environ. Sci. Technol.

[34] Cerri M., Wille F., Arn S., Bucheli T. D., Widmer F., Werz R., McNeill K., Manfrin A., Sander M. (2025). An Analytical
Workflow to Quantify Biodegradable Polyesters in Soils and Its Application
to Incubation Experiments. Environ. Sci. Technol.

[35] Lindström A., Albertsson A. C., Hakkarainen M. (2004). Quantitative
Determination of Degradation
Products an Effective Means to Study Early Stages of Degradation in
Linear and Branched Poly­(Butylene Adipate) and Poly­(Butylene Succinate). Polym. Degrad. Stab.

[36] East G. C., Girshab A. M. (1982). Cyclic Oligomers
in Poly­(1,4-Butylene Terephthalate). Polymer.

[37] Zumstein M. T., Schintlmeister A., Nelson T. F., Baumgartner R., Woebken D., Wagner M., Kohler H.-P. E., McNeill K., Sander M. (2018). Biodegradation of Synthetic Polymers in Soils: Tracking
Carbon into CO_2_ and Microbial Biomass. Sci. Adv.

[38] Sorensen L. H. (1975). The Influence
of Clay on the Rate of Decay of Amino Acid Metabolites Synthesized
in Soils during Decomposition of Cellulose. Soil Biol. Biochem.

[39] Sorensen L. H. (1981). Carbon-Nitrogen
Relationships during the Humification of Cellulose in Soils Containing
Different Amounts of Clay. Soil Biol. Biochem.

[40] Manzoni S., Taylor P., Richter A., Porporato A., Ågren G. I. (2012). Environmental and Stoichiometric Controls on Microbial
Carbon-Use Efficiency in Soils. New Phytol.

[41] Sinsabaugh R. L., Turner B. L., Talbot J. M., Waring B. G., Powers J. S., Kuske C. R., Moorhead D. L., Follstad Shah J. J. (2016). Stoichiometry
of Microbial Carbon Use Efficiency in Soils. Ecol. Monogr.

[42] Qiao Y., Wang J., Liang G., Du Z., Zhou J., Zhu C., Huang K., Zhou X., Luo Y., Yan L., Xia J. (2019). Global Variation of Soil Microbial Carbon-Use Efficiency in Relation
to Growth Temperature and Substrate Supply. Sci. Rep.

[43] Witt U., Müller R. J., Deckwer W. D. (1997). Biodegradation Behavior and Material
Properties of Aliphatic/Aromatic Polyesters of Commercial Importance. J. Environ. Polym. Degrad.

[44] Zumstein M. T., Narayan R., Kohler H.-P. E., McNeill K., Sander M. (2019). Dos and Do
Nots When Assessing the Biodegradation of Plastics. Environ. Sci. Technol.

[45] Sander M. (2019). Biodegradation
of Polymeric Mulch Films in Agricultural Soils: Concepts, Knowledge
Gaps, and Future Research Directions. Environ.
Sci. Technol.

[46] Kijchavengkul T., Auras R., Rubino M., Selke S., Ngouajio M., Thomas Fernandez R. (2010). Biodegradation and Hydrolysis Rate of Aliphatic Aromatic
Polyester. Polym. Degrad. Stab.

[47] Rychter P., Kawalec M., Sobota M., Kurcok P., Kowalczuk M. (2010). Study of Aliphatic-Aromatic
Copolyester Degradation in Sandy Soil and Its Ecotoxicological Impact. Biomacromolecules.

[48] Convertino F., Carroccio S. C., Cocca M. C., Dattilo S., Dell’acqua A. C., Gargiulo L., Nizzetto L., Riccobene P. M., Schettini E., Vox G., Zannini D., Cerruti P. (2024). The Fate of
Post-Use Biodegradable PBAT-Based Mulch Films Buried in Agricultural
Soil. Sci. Total Environ.

[49] Hoshino A., Sawada H., Yokota M., Tsuji M., Fukuda K., Kimura M. (2001). Influence of Weather
Conditions and Soil Properties
on Degradation of Biodegradable Plastics in Soil. Soil Sci. Plant Nutr.

[50] Yamamoto-Tamura K., Hiradate S., Watanabe T., Koitabashi M., Sameshima-Yamashita Y., Yarimizu T., Kitamoto H. (2015). Contribution
of Soil
Esterase to Biodegradation of Aliphatic Polyester Agricultural Mulch
Film in Cultivated Soils. AMB Express.

[51] Fu Y., Wu G., Bian X., Zeng J., Weng Y. (2020). Biodegradation Behavior
of Poly­(Butylene Adipate-Co-Terephthalate) (PBAT), Poly­(Lactic Acid)
(PLA), and Their Blend in Freshwater with Sediment. Molecules.

[52] Wohlleben W., Rückel M., Meyer L., Pfohl P., Battagliarin G., Hüffer T., Zumstein M., Hofmann T. (2023). Fragmentation
and Mineralization
of a Compostable Aromatic–Aliphatic Polyester during Industrial
Composting. Environ. Sci. Technol. Lett.

