# Supplementary material for: Biodegradation of Synthetic Aliphatic-Aromatic Polyesters in Soils: Linking Chemical Structure to Biodegradability
Source: Environ Sci Technol. 2025 Sep 12;59(37):19966–77. doi: 10.1021/acs.est.5c03099 (PMC12461927; doi:10.1021/acs.est.5c03099)
Supplement: Supplementary file 1 [file es5c03099_si_001.pdf]

Supporting Information

Biodegradation of synthetic aliphatic-aromatic polyesters in soils: linking chemical structure to biodegradability

Taylor F. Nelson[1], Rebekka Baumgartner[1], Madalina Jaggi[2], Stefano Bernasconi[2], Glauco Battagliarin[3], Carsten Sinkel[3], Andreas Künkel[3], Hans-Peter E. Kohler[4], Kristopher McNeill[1], and Michael Sander[1*]

[1] Institute of Biogeochemistry and Pollutant Dynamics, ETH Zurich, 8092 Zurich, Switzerland

[2] Geological Institute, Department of Earth Sciences, ETH Zurich, 8092 Zurich, Switzerland

[3] BASF SE, Carl-Bosch-Strasse 38, Ludwigshafen 67056, Germany

[4] Environmental Biochemistry Group; Environmental Microbiology, Swiss Federal Institute of Aquatic Science and Technology (Eawag), 8600 Dübendorf, Switzerland

Number of pages: 19

Number of figures: 4

Number of tables: 2

S1. Physicochemical properties of polyesters

The monomer compositions, carbon isotopic compositions, molecular weights, and key thermal properties of the used ^{13}C-labelled polyesters are given in **Table S1**.

We determined the carbon isotopic signatures, $\delta^{13}C_{polyester}$ (‰), of the synthesized ^{13}C-labelled polyesters using elemental analysis coupled to isotope ratio mass spectrometry (EA-IRMS). These signatures were referenced to the ^{13}C content of the ^{13}C-standard Vienna Pee Dee Belemnite (VPDB) according to Eq. S1:

$$\delta^{13}C_{polyester} = \left(\frac{(^{13}C/^{12}C)_{polyester}}{(^{13}C/^{12}C)_{VPDB}} - 1\right) \cdot 1000 \qquad \text{(Eq. S1)}$$

where $(^{13}C/^{12}C)_{VPDB}$ is the carbon isotope ratio of VPDB (= 0.0112372). We then calculated the ^{13}C atom% of each ^{13}C-labelled polyester, $\%^{13}C_{polyester}$, using the carbon isotope ratio of the polyester, $(^{13}C/^{12}C)_{polyester}$ (obtained from Eq. S1), according to Eq. S2:

$$\%^{13}C_{polyester} = \frac{(^{13}C/^{12}C)_{polyester}}{1 + (^{13}C/^{12}C)_{polyester}} \qquad \text{(Eq. S2)}$$

Table S1. Physicochemical properties of ^{13}C-labelled aliphatic-aromatic co-polyesters used in soil incubations. [a]

polymer short name	labelled monomer	monomeric composition [b]		T content [c]	$\delta^{13}C$ [d]	atom% ^{13}C [e]	$M_n;M_w$ [f]	$T_g;T_m$ [g]
		monomers	mol %	mol% of total diacids	‰	%	Da	°C
$P(^{13}C_4\text{-}B)A_{50}T_{50}$	$^{13}C_4\text{-}B$	B	49.9	49.5	2’285	3.56	11’700; 38’300	-37; 123
		A	25.3					
		T	24.8					
$PB(1,6\text{-}^{13}C_2\text{-}A)_{50}T_{50}$	$1,6\text{-}^{13}C_2\text{-}A$	B	49.6	49.4	2’472	3.76	14’320; 37’830	-37; 126
		A	25.5					
		T	24.9					
$PBA_{50}(1\text{-}^{13}C_1\text{-}T)_{50}$	$1\text{-}^{13}C_1\text{-}T$	B	50.1	50.1	2’258	3.53	15’160; 37’260	-38; 123
		A	24.9					
		T	25.0					
$P(^{13}C_4\text{-}B)Se_{50}T_{50}$	$^{13}C_4\text{-}B$	B	49.6	49.8	2’274	3.55	17’150; 43’400	-41; 121
		Se	25.3					
		T	25.1					
$PBSe_{50}(1\text{-}^{13}C_1\text{-}T)_{50}$	$1\text{-}^{13}C_1\text{-}T$	B	50.1	49.7	2’286	3.56	14’950; 39’080	-41; 121
		Se	25.1					
		T	24.8					
$P(^{13}C_4\text{-}B)A_{100}T_0$	$^{13}C_4\text{-}B$	B	50.0	0	2’674	3.97	16’700 52’800	-59; 56
		A	50.0					
$P(^{13}C_4\text{-}B)A_{80}T_{20}$	$^{13}C_4\text{-}B$	B	50.0	19.4	2’272	3.55	15’800 46’700	-45; 40
		A	40.3					
		T	9.7					
$P(^{13}C_4\text{-}B)A_{70}T_{30}$	$^{13}C_4\text{-}B$	B	50.0	30.0	2’254	3.53	15’800 44’600	-43; 62
		A	35.0					
		T	15.0					
$P(^{13}C_4\text{-}B)A_{53}T_{47}$	$^{13}C_4\text{-}B$	B	50.0	46.8	2’272	3.55	14’600 41’300	-36; 119
		A	26.6					
		T	23.4					

[a] properties of $P(^{13}C_4\text{-}B)A_{50}T_{50}$, $PB(1,6\text{-}^{13}C_2\text{-}A)_{50}T_{50}$, $PBA_{50}(1\text{-}^{13}C_1\text{-}T)_{50}$ were also given in a previous publication[1]

[b] mol% of monomeric units were determined by relative peak areas of monomeric unit-specific peaks in the polyester ^{1}H-NMR spectra

[c] T content = % T of total diacids, i.e., mol% T / (mol% A or Se + mol% T) from monomeric compositions

[d] $\delta^{13}C$ values measured using EA-IRMS

[e] atom% ^{13}C calculated from measured $\delta^{13}C$ values using equations Eq. S1 and Eq. S2

[f] M_n and M_w = polyester number-average and weight-average molecular weight, respectively, determined using gel permeation chromatography vs PMMA calibration standards and HFIP with 0.05% KTFAc as a solvent, operated at 40 °C with a flow rate of 1 mL min^{-1} (see further detail below)

[g] T_g and T_m = polyester glass-transition and melting temperatures, respectively, determined using differential scanning calorimetry

Detailed information on the SEC method. The molar mass distributions of the polymers were determined by Size Exclusion Chromatography (SEC). A modular SEC system from Agilent (1100 and 1200 series) with a combination of columns (Shodex HFIP-LG Guard column and 2 x PL HFIPgel columns, 300 x 7.5 mm, 3-100 µm from Agilent) was equipped with a refractive index detector. The column temperature was set to 40°C and the eluent flow rate of HFIP (1,1,1,3,3,3 – Hexafluoro-2-propanol) with 0,05% KTFAc was 1.0 mL/min. Calibration was conducted using a polymethylmethacrylate calibration kit (PSS Polymer Standards Service GmbH) between 800 g/mo and 1 820 000 g/mol. The samples were dissolved in the eluent at a concentration of approx. 1.5 mg/mL and filtered through Millipore-Millex-FG 0.2 µm filters. Of these solutions, 50 µL were injected per sample.

S2. Calculations of polymer mineralization rates and extents.

These calculations were performed as previously described,[2] and identically for incubations containing polyester and cellulose, both of which we refer to inclusively as 'polymer' in the calculations below. To quantify the extents of polyester and cellulose mineralization over time, we first calculated the fractional contribution of carbon from the added polymer to the total measured soil efflux CO_2 concentrations by using the ^{13}C atom percentages of the efflux CO_2 from soils with added polymer, $\%^{13}C_{soil+polymer}$, without added polymer, $\%^{13}C_{soil}$, and of the polymer material itself, $\%^{13}C_{polymer}$:[3]

$$f_{\text{polymer}} = \frac{(\%^{13}\text{C}_{\text{soil+polymer}} - \%^{13}\text{C}_{\text{soil}})}{(\%^{13}\text{C}_{\text{polymer}} - \%^{13}\text{C}_{\text{soil}})} \qquad \text{(Eq. S3)}$$

Based on $f_{polymer}$ and the ^{13}C atom% of the added polymer, $\%^{13}C_{polymer}$, we calculated the concentration of the total measured soil efflux CO_2, $[CO_2]_{soil+polymer}$ (ppm), that was derived from polymer-^{13}C, $[^{13}CO_2]_{polymer}$ (ppm):

$$[^{13}\text{CO}_2]_{\text{polymer}} = f_{\text{polymer}} \cdot [\text{CO}_2]_{\text{soil+polymer}} \cdot \%^{13}\text{C}_{\text{polymer}} \qquad \text{(Eq. S4)}$$

The rate of polymer-^{13}C mineralization, $r(^{13}C_{mineralized})$ (µg ^{13}C h^{-1}), at any given time per bottle was calculated using the volumetric flow rate of gas through the incubation bottles (Q = 1.44 L h^{-1}), the molar mass of ^{13}C (M = 13.003 g mol^{-1}), and the molar volume of air assuming 25 °C and 1 atm (V = 24.465 L mol^{-1}):

$$r(^{13}\text{C}_{\text{mineralized}}) = {}^{13}[\text{CO}_2]_{\text{polymer}} \cdot \frac{\text{Q} \cdot \text{M}}{\text{V}} \qquad \text{(Eq. S5)}$$

Finally, we integrated the polymer-^{13}C mineralization rate over the incubation time t (h), to obtain the cumulative amount of polymer-^{13}C mineralization, and normalized this to the amount of polymer-^{13}C added to each incubation bottle, $n(^{13}C_{added})$ (µg ^{13}C), yielding the cumulative percent of the added polymer-^{13}C that had mineralized at each time, $^{13}C_{mineralized}$ (%):

$$^{13}C_{\text{mineralized}} = \frac{\int_0^t r(^{13}C_{\text{mineralized}})\, dt}{n(^{13}C_{\text{added}})} \cdot 100 = \frac{n(^{13}C_{\text{mineralized}})}{n(^{13}C_{\text{added}})} \cdot 100 \qquad \text{(Eq. S6)}$$

S3. Details on quantifying total non-mineralized polyester- and cellulose-added ^{13}C remaining in soils, $^{13}C_{non-mineralized}$.

S3-1. Homogenization of soil samples prior to EA-IRMS analysis

In brief, after freeze-drying and milling (see Materials and Methods section of the main text), a 3 g subsample of each soil was suspended in chloroform and pulse sonicated on ice (0.8 seconds on / 0.3 seconds off; 500 W output, 40 % max. amplitude) using an ultrasonic processor equipped with a tapered microtip probe (Sonics; 1 mm tip diameter). Following removal of the chloroform at ambient temperature and pressure in a ventilated hood overnight, the soils were placed under a vacuum (0.01 mbar) for at least 24 hours to ensure complete removal of residual chloroform.

S3-2. Calibration of the EA-IRMS

Several organic standard compounds with known $\delta^{13}C$ were used for instrument calibration (i.e., nicotinamide (Thermo), %C = 59.0, $\delta^{13}C$ = -31.2 ‰; peptone (Sigma-Aldrich), %C = 43.4, $\delta^{13}C$ = -15.6 ‰; glucose (custom mixture), %C = 40.0, $\delta^{13}C$ = 60.9 ‰)). The ^{13}C-enriched glucose standard was prepared by mixing non-labelled glucose (Sigma-Aldrich) with $^{13}C_6$-glucose (labelling extent 24-25 %; Cambridge Isotope Labs) via bead beating with zirconia beads to obtain a standard with a reproducible and high $\delta^{13}C$ (= 60.7 (± 0.7) ‰; mean ± standard deviation of 18 replicates), as previously reported.[2]

S3-3. Calculating non-mineralized polymer-derived carbon in soils

As for $^{13}C_{mineralized}$, these calculations were performed identically for soils containing polyesters or cellulose, which are both referred to below as 'polymer'. To quantify $^{13}C_{non-mineralized}$, we first calculated the fractional contribution of carbon from the added polymers to the total measured soil carbon by using Equation S3, where $\%^{13}C_{soil+polymer}$, $\%^{13}C_{soil}$, and $\%^{13}C_{polymer}$ refer

to the ^{13}C atom% of CO_2 resulting from combustion of soils with added polymer, soils without added polymer, and of only the polymer, respectively.

For each sample, we then calculated the mass of non-mineralized polymer-derived ^{13}C, $n(^{13}C_{non\text{-}mineralized})$ (µg ^{13}C), by multiplying the total carbon of the analyzed aliquot, $n(C_{soil+polymer})$ (µg C), by $f_{polymer}$ and the ^{13}C atom% of the added polymer, $\%^{13}C_{polymer}$, as well as the ratio of the total soil mass in the incubation, $m(soil_{incubation})$ (g soil) to the mass of the soil aliquot analyzed using EA-IRMS, $m(soil_{EA\text{-}IRMS})$ (g soil):

$$n(^{13}C_{non-mineralized}) = n(C_{soil+polymer}) \cdot f_{polymer} \cdot \%^{13}C_{polymer} \cdot \frac{m(soil_{incubation})}{m(soil_{EA-IRMS})} \quad \text{(Eq. S7)}$$

We normalized $n(^{13}C_{non\text{-}mineralized})$ to the amount of polymer-^{13}C added to each incubation bottle, $n(^{13}C_{added})$ (µg ^{13}C), to obtain the percent of the added polymer-^{13}C that was non-mineralized in the soils at the end of the incubations, $^{13}C_{non\text{-}mineralized}$ (%):

$$^{13}C_{\ non-mineralized} = \frac{n(^{13}C_{non-mineralized})}{n(^{13}C_{added})} \cdot 100 \quad \text{(Eq. S8)}$$

S4. Details on quantifying residual polyester in soils, $^{13}C_{polymer\ residual}$.

S4-1. NMR operation

We used the following values for key acquisition parameters: P1 (applied pulse length) = 14 µs, NS (number of acquisition scans) = 128, DS (number of dummy scans) = 16, D1 (delay time between scans) = 15 s. We processed the acquired ^1H-NMR spectra using the software MNova (MestreLab), by (i) referencing the chemical shifts (δ; ppm) to the peak of residual non-deuterated chloroform ($CHCl_3$; δ = 7.26 ppm), (ii) performing manual phase shift corrections, and (iii) performing automatic baseline corrections using a Bernstein polynomial. We then manually integrated the spectra peaks used for polyester quantification (see **Figure S1** for peak assignments

to spectra of representative polyesters, and for recoveries of these polyesters spiked into soil using the extraction method). All peaks at chemical shifts further up-field in the spectra (i.e., < 3 ppm) tended to show stronger interference with background noise presumably caused by residual solvents or co-extracted soil organic matter. These peaks were thus not used for polyester quantification.

S4-2. NMR quantification of residual polyester

Calculations to determine polyester amounts in extracts. As the peaks from aryl ^{1}H were the most well-resolved of the spectra, we primarily used their areas for quantification of polyesters in the extracts. However, the terephthalate (T) content of the polyester in each extract had to be determined, given that each polyester variant had a slightly different initial T content, and the T contents were subject to change by the end of the incubation due to preferential biodegradation of aliphatic-rich polyester segments. Therefore, to convert from determined terephthalate amount in each extract to mass of bulk polyester, we first calculated the %T and %A or %Se of the extracted polyester (i.e., % of diacid monomer units that are terephthalate, or adipate, or sebacate, respectively). For this, we used the peak areas for polyester butylene ^{1}H adjacent to T units, A_{B_1} (δ = 4.40 ppm), and those adjacent to A or Se units in PBAT and PBSeT, respectively, A_{B_2} (δ = 4.12 ppm), as these peaks correspond to structurally similar protons and are located in the same spectral region, according to:

$$\%T = \frac{A_{B_1}}{A_{B_1} + A_{B_2}} \cdot 100 \qquad \text{(Eq. S9a)}$$

$$\%A\ (\text{or } \%\ Se) = \frac{A_{B_2}}{A_{B_1} + A_{B_2}} \cdot 100 \qquad \text{(Eq. S9b)}$$

We then calculated the moles of terephthalate in each extract, $n_{\text{terephthalate}}$ (*mol*), based on the ratio of peak areas from the terephthalate-monomer unit aryl ^{1}H, A_T (δ = 8.09 ppm), to DMB aryl ^{1}H,

A_{DMB_1} (δ = 6.89 ppm), the known mass of DMB we added, m_{DMB} (g), and its molecular weight, M_{DMB} (= 138.17 g mol^{-1}):

$$n_{\mathrm{terephthalate}} = \frac{A_{\mathrm{T}}}{A_{\mathrm{DMB}_1}} \cdot \frac{m_{\mathrm{DMB}}}{M_{\mathrm{DMB}}} \quad \text{(Eq. S10)}$$

From the moles of terephthalatic acid units in each extract, $n_{\mathrm{terephthalate}}$, we then calculated the mass of bulk polyester extracted, $m_{\mathrm{polyester}}$ (g), using the molecular weight of a -BT- repeat unit, M_{BT} (= 220.22 g mol^{-1}), and a -BA- repeat unit, M_{BA} (= 200.23 g mol^{-1}), corrected for the actual ratio of BA:BT monomer units in the extracted polyester calculated in Eq. S9a & S9b:

$$m_{\mathrm{polyester}} = n_{\mathrm{terephthalate}} \cdot (M_{\mathrm{BT}} + M_{\mathrm{BA}} \frac{\%\mathrm{A}}{\%\mathrm{T}}) \quad \text{(Eq. S11)}$$

The same calculations were performed to calculate masses of PBSeT extracted, replacing %A in Eq. S9b & S11 with %Se (using the "B_1" and "B_2" peaks at the same chemical shifts in the corresponding spectra) and M_{BA} in Eq. S11 with M_{BSe} (= 256.34 g mol^{-1}). For calculating extracted amounts of $PBA_{100}T_0$ that contained no T (i.e., PBA), we instead used the peak area ratios of polyester-B ^{1}H, A_{B_2} (δ = 4.09 ppm), to DMB-methoxy ^{1}H, A_{DMB_2} (δ = 3.77 ppm), and M_{BA}:

$$m_{\mathrm{PBA100T0}} = \frac{A_{\mathrm{B}_2}}{A_{\mathrm{DMB}_2}} \cdot \frac{m_{\mathrm{DMB}}}{M_{\mathrm{DMB}}} \cdot M_{\mathrm{BA}} \quad \text{(Eq. S12)}$$

Finally, we normalized the mass of polyester in each extract to the mass of polyester added to the incubation, m_{added} (g), and multiplied by the ratio of soil mass in the incubation, $m_{\mathrm{soil,\ incubated}}$ (g), to soil mass used for extraction, $m_{\mathrm{soil,\ extracted}}$ (g), to obtain the relative amount of residual to added polyester in the soil at the end of the incubation, $^{13}C_{\mathrm{polyester\ residual}}$ (%):

$$^{13}\mathrm{C}_{\mathrm{polyester\ residual}} = \frac{m_{\mathrm{polyester}}}{m_{\mathrm{added}}} \cdot \frac{m_{\mathrm{soil,\ incubated}}}{m_{\mathrm{soil,\ extracted}}} \cdot 100 \quad \text{(Eq. S13)}$$

S4-3. Reference ^{1}H-NMR spectra and extraction recoveries of polyesters

Figure S1 shows example ^{1}H-NMR spectra of representative polyesters (i.e., polybutylene adipate-*co*-terephthalate (PBAT) with different T contents, $PBA_{50}T_{50}$, $PBA_{80}T_{20}$, and $PBA_{100}T_0$

(i.e., PBA); and polybutylene sebacate-*co*-terephthalate ($PBSe_{52}T_{48}$)), together with the internal standard 1,4-dimethoxybenze (DMB) used for polyester quantification by q-^{1}H-NMR. Note that these polyesters were not ^{13}C-labelled, but were synthesized in the same manner as the ^{13}C-labelled polyesters used in the incubation experiments only using non-labelled monomers. We therefore used these non-labelled polyester variants for the validation of the extraction and quantification methods previously developed for $PBA_{50}T_{50}$,[4] when applied to the other polyester variants used in this study. We prepared all samples shown in **Figure S1** by dissolving 3 mg polyester in 1 mL deuterated chloroform ($CDCl_3$) containing 1 mg DMB mL^{-1} as an internal quantification standard. We then acquired ^{1}H-NMR spectra using the acquisition parameters stated above in section S4-1. These spectra demonstrate adequate resolution of peaks assigned to polyesters and DMB ^{1}H which were used for determination of polyester T contents and for their quantification in soil extracts.

We used spike-recovery experiments to test the recoveries of these representative polyesters from the soil used in incubation experiments. Polyesters were added to freeze-dried soils in known amounts by syringe-transferring defined volumes of polyester concentration solutions in $CHCl_3$. After evaporation of the $CHCl_3$, the soils were Soxhlet extracted, followed by quantification of polyesters in the extracts, according to the protocols detailed above and in the Materials and Methods section of the main manuscript. All polyesters were found to be quantitatively extracted from the tested soil, with duplicate extraction samples per polyester yielding recoveries of 99.2 and 100.2 %, 96.7 and 96.8 %, and 100.2 and 100.7 % of the added polyester for $PBA_{80}T_{20}$, PBA, and $PBSe_{52}T_{48}$, respectively. We have previously demonstrated the quantitative extraction of $PBA_{50}T_{50}$ from different soils, including the soil used herein for incubations.[4]

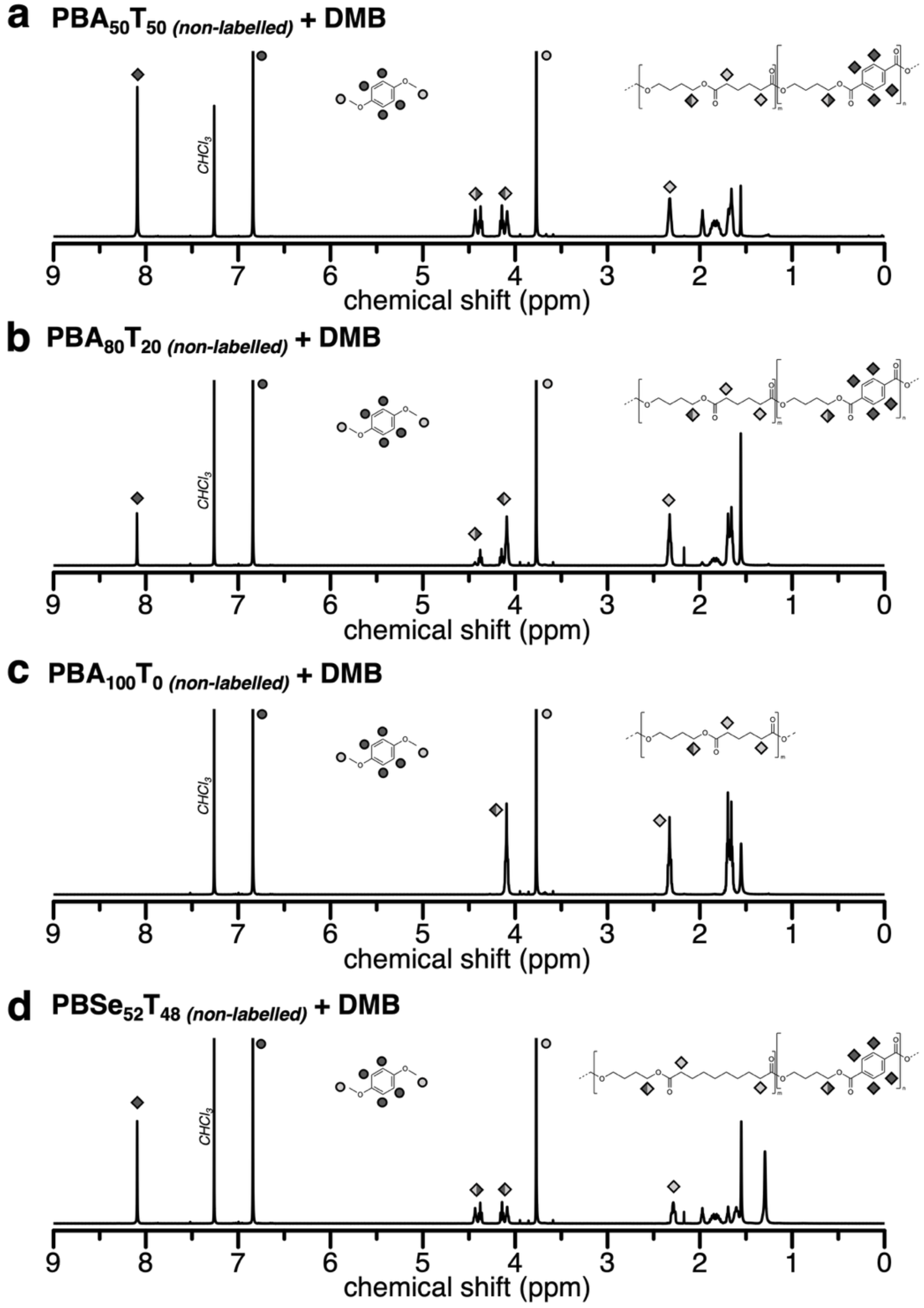

Figure S1. ^{1}H-NMR spectra and chemical structures of different aliphatic-aromatic co-polyesters (3 mg) dissolved in 1 mL deuterated chloroform ($CDCl_3$) containing 1 mg of the internal quantification standard 1,4-dimethoxybenzene (DMB). Note that these polyesters are non-labelled variants, used for the validation of the extraction and quantification methods. **a**: polybutylene adipate-*co*-terephthalate (PBAT) with a T content of 50%, $PBA_{50}T_{50}$; **b**: $PBA_{80}T_{20}$ with a T content of 20%; **c**: polybutylene adipate (PBA), i.e., '$PBA_{100}T_0$' with a T content of 0%; **d**: polybutylene sebacate-*co*-terephthalate (PBSeT) with a T content of 48%, $PBSe_{52}T_{48}$. Symbols match ^{1}H-NMR spectral peaks with the corresponding ^{1}H attached to the carbons in the structure shown for each panel.

S5. Mineralization dynamics of P($^{13}C_4$-B)$A_{100\text{-}X}T_X$ in soil

Figure S2 shows measured mineralization rates of PB$A_{100\text{-}X}T_X$ variants with different T contents (all ^{13}C-labelled in the butanediol monomeric unit) during incubation. The rate data is replotted from **Figure 2e**; in **Figure S2**, we show ^{13}C-mineralization rates values ≤ 0.3 μg ^{13}C h^{-1} to visualize the mineralization rates also for the slowly mineralizing (relative to the other variants) P($^{13}C_4$-B)$A_{53}T_{47}$ variant. This representation shows that throughout the incubation, mineralization rates were above zero even for this slowly mineralizing variant.

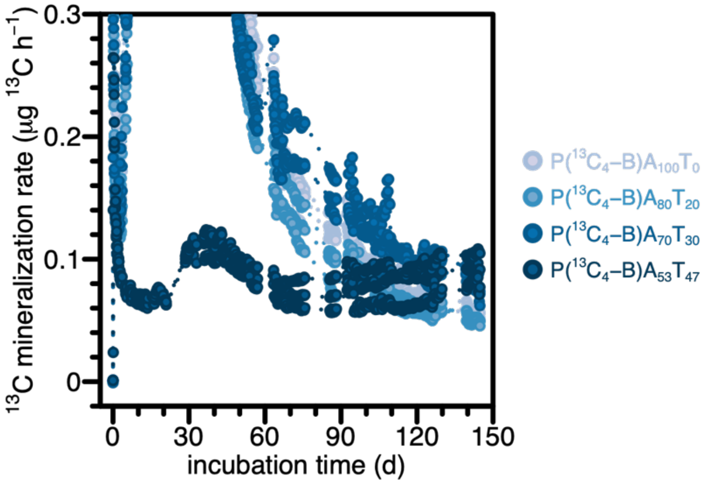

Figure S2. Measured polyester-^{13}C mineralization rates of butanediol-^{13}C-labelled variants of polybutylene adipate-*co*-terephthalate (PBAT) with different ratios of diacid monomer-units (i.e., P($^{13}C_4$-B)$A_{100\text{-}X}T_X$, where X represents the mol percent of terephthalate units to the total diacid units). Triplicate soil incubations bottles were followed for each variant up to 145 days of incubation, and measured points are plotted for each individual replicate, with dashed lines representing linear interpolations between measured points. Data is replotted from **Figure 2e** to show the slow but non-zero mineralization rates of P($^{13}C_4$-B)$A_{53}T_{47}$ throughout the entire incubation.

S6. Mineralization rates of cellulose-^{13}C vs. total cellulose-^{13}C added to soil

Figure S3 shows maximum measured mineralization rates of cellulose-^{13}C in soil incubations vs. the total amounts of cellulose-^{13}C added to each incubation. Maximum ^{13}C-mineralization rates were taken from data plotted in **Figure 2g** (shown in the inset) and total cellulose-^{13}C added amounts were calculated assuming 99% and 1.1% (i.e., natural abundance) ^{13}C-contents for 'labelled' and 'non-labelled' cellulose, respectively, which were mixed in different mass ratios prior to adding to soil.

The maximum cellulose-^{13}C mineralization rates showed an approximately linear dependence on the amount of cellulose-^{13}C added (with a rate constant of 0.0051 h^{-1}) with a good fit when forced through the origin ($R^2 = 0.97$). The finding of a linear relationship suggests that the two cellulose pools mineralized with indistinguishable rates.

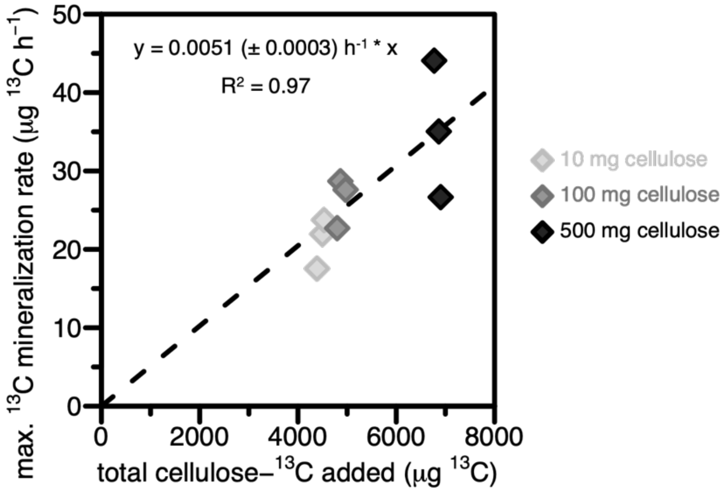

Figure S3. Measured maximum mineralization rates of ^{13}C-labelled cellulose in soil vs. total amounts of cellulose-^{13}C added to the soil at the onset of the incubations. Different amounts of cellulose were prepared by mixing ^{13}C-labelled and non-labelled cellulose in different mass ratios, resulting in different total amounts of ^{13}C-cellulose added. The data points represent individual triplicate incubations per added cellulose amount. The dashed line indicates the linear least squares regression fit forced through the origin, with the corresponding equation and R^2-value provided.

S7. Compilation of $^{13}C_{mineralized}$, $^{13}C_{non\text{-}mineralized}$, and $^{13}C_{polymer\ residual}$ data for all incubations

The quantified values of $^{13}C_{mineralized}$, $^{13}C_{non\text{-}mineralized}$, and $^{13}C_{polymer\ residual}$, and calculated values for $^{13}C_{biomass}$, as well as values of the T contents for residual, extracted polyesters, for each polymer incubation experiment are compiled in **Table S2**. The data is also shown in **Figures 2**, **3** and **4** in the main manuscript. The mass balance data (including both $^{13}C_{minearlized}$ and $^{13}C_{non\text{-}mineralized}$) for 100 mg of added cellulose has been published previously.[2]

Table S2. Compilation of ^{13}C-labelled polymer biodegradation results for all individual soil incubation bottles.

Polymer	Soil	Rep. #	Incubation time (d)	$^{13}C_{mineralized}$	$^{13}C_{non\text{-}mineralized}$	$^{13}C_{polymer\ residual}$	$^{13}C_{biomass}$	T content of residual (T/(T + A or Se) (%))
				(% of added polymer-$^{(13)}$C)				
Position-specifically ^{13}C-labelled PBAT								
$PBA_{50}(1\text{-}^{13}C_1\text{-}T)_{50}$	LiHof 1	1	319	21.0	74.8	75.6	-0.8	50.1
$PBA_{50}(1\text{-}^{13}C_1\text{-}T)_{50}$	LiHof 1	2	425	26.7	71.6	68.2	3.4	51.0
$PBA_{50}(1\text{-}^{13}C_1\text{-}T)_{50}$	LiHof 1	3	425	26.2	75.1	65.4	9.7	51.1
$PB(1,6\text{-}^{13}C_2\text{-}A)_{50}T_{50}$	LiHof 1	1	319	86.2	13.6	14.1	-0.5	56.2
$PB(1,6\text{-}^{13}C_2\text{-}A)_{50}T_{50}$	LiHof 1	2	425	26.0	84.2	64.1	20.1	50.1
$PB(1,6\text{-}^{13}C_2\text{-}A)_{50}T_{50}$	LiHof 1	3	425	23.0	75.1	78.5	-3.5	49.7
$P(^{13}C_4\text{-}B)A_{50}T_{50}$	LiHof 1	1	319	15.1	84.5	78.5	6.0	49.5
$P(^{13}C_4\text{-}B)A_{50}T_{50}$	LiHof 1	2	425	17.8	77.3	78.5	-1.2	49.5
$P(^{13}C_4\text{-}B)A_{50}T_{50}$	LiHof 1	3	425	17.3	79.3	70.3	9.1	49.4
Position-specifically ^{13}C-labelled PBSeT								
$P(^{13}C_4\text{-}B)Se_{50}T_{50}$	LiHof 1	1	319	66.9	39.4	21.9	17.5	57.9
$P(^{13}C_4\text{-}B)Se_{50}T_{50}$	LiHof 1	2	425	56.5	42.8	35.8	7.0	61.4
$P(^{13}C_4\text{-}B)Se_{50}T_{50}$	LiHof 1	3	425	59.9	43.9	21.7	22.2	57.1
$PBSe_{50}(1\text{-}^{13}C_1\text{-}T)_{50}$	LiHof 1	1	319	46.2	56.9	36.9	20.1	59.1
$PBSe_{50}(1\text{-}^{13}C_1\text{-}T)_{50}$	LiHof 1	2	425	52.2	55.5	29.8	25.7	59.2
$PBSe_{50}(1\text{-}^{13}C_1\text{-}T)_{50}$	LiHof 1	3	425	51.7	51.4	30.3	21.1	60.5
PBAT with varying T content								
$P(^{13}C_4\text{-}B)A_{100}T_0$	LiHof 2	1	145	79.3	20.0	2.3	17.7	0.0
$P(^{13}C_4\text{-}B)A_{100}T_0$	LiHof 2	2	145	83.8	19.9	1.8	18.1	0.0
$P(^{13}C_4\text{-}B)A_{100}T_0$	LiHof 2	3	145	81.6	20.2	3.2	17.0	0.0
$P(^{13}C_4\text{-}B)A_{100}T_{20}$	LiHof 2	1	145	72.2	19.7	1.9	17.9	28.6
$P(^{13}C_4\text{-}B)A_{80}T_{20}$	LiHof 2	2	145	83.3	20.3	1.7	18.6	27.6
$P(^{13}C_4\text{-}B)A_{80}T_{20}$	LiHof 2	3	145	77.1	19.5	2.3	17.2	31.2
$P(^{13}C_4\text{-}B)A_{80}T_{30}$	LiHof 2	1	145	73.8	27.7	10.4	17.3	34.7
$P(^{13}C_4\text{-}B)A_{80}T_{30}$	LiHof 2	2	145	76.0	26.8	10.6	16.2	36.7
$P(^{13}C_4\text{-}B)A_{80}T_{30}$	LiHof 2	3	145	75.0	25.7	10.3	15.4	34.4
$P(^{13}C_4\text{-}B)A_{53}T_{47}$	LiHof 2	1	145	14.6	91.2	88.0	3.2	47.5
$P(^{13}C_4\text{-}B)A_{53}T_{47}$	LiHof 2	2	145	11.6	85.5	89.0	-3.5	46.9
$P(^{13}C_4\text{-}B)A_{53}T_{47}$	LiHof 2	3	145	13.3	83.3	96.8	-13.5	47.3
Different amounts of U-$^{13}C_6$-cellulose								
cellulose (500 mg)	LiHof 2	1	139	78.8	17.6	N/A	N/A	N/A
cellulose (500 mg)	LiHof 2	2	254	81.4	16.3	N/A	N/A	N/A
cellulose (500 mg)	LiHof 2	3	254	83.8	14.7	N/A	N/A	N/A
cellulose (100 mg)	LiHof 2	1	139	73.6	23.5	N/A	N/A	N/A
cellulose (100 mg)	LiHof 2	2	254	75.9	22.3	N/A	N/A	N/A
cellulose (100 mg)	LiHof 2	3	254	75.3	22.3	N/A	N/A	N/A
cellulose (10 mg)	LiHof 2	1	139	70.2	24.9	N/A	N/A	N/A
cellulose (10 mg)	LiHof 2	2	254	78.9	24.4	N/A	N/A	N/A
cellulose (10 mg)	LiHof 2	3	254	77.2	22.0	N/A	N/A	N/A

Section S8. Correlation between $^{13}C_{polymer\ residual}$ and $^{13}C_{non\text{-}mineralized}$ for polyesters at the end of soil incubations

Figure S4 shows the measured values of $^{13}C_{polymer\ residual}$ determined at the end of soil incubations vs. $^{13}C_{non\text{-}mineralized}$ for the different ^{13}C-labelled polyester variants. $^{13}C_{polymer\ residual}$ values increased with increasing $^{13}C_{non\text{-}mineralized}$. As indicated in **Figure 3** of the main text, values of $^{13}C_{non\text{-}mineralized}$ are indirectly validated by closing of mass balances on polyester-added ^{13}C (when combined with measured of $^{13}C_{mineralized}$). As discussed in the main text, variabilities in measured $^{13}C_{polymer\ residual}$ (and therefore variability around the 1:1 line of **Figure S4**) likely reflected that the subsamples extracted were not fully representative of the respective total amount of soil incubated, consistent with residual polyesters being heterogeneously distributed in the soils. Deviations from the 1:1 line observed for PBSeT as well as for PBAT variants with lower T contents reflect higher incorporations of polyester-added ^{13}C into microbial biomass ($^{13}C_{biomass}$).

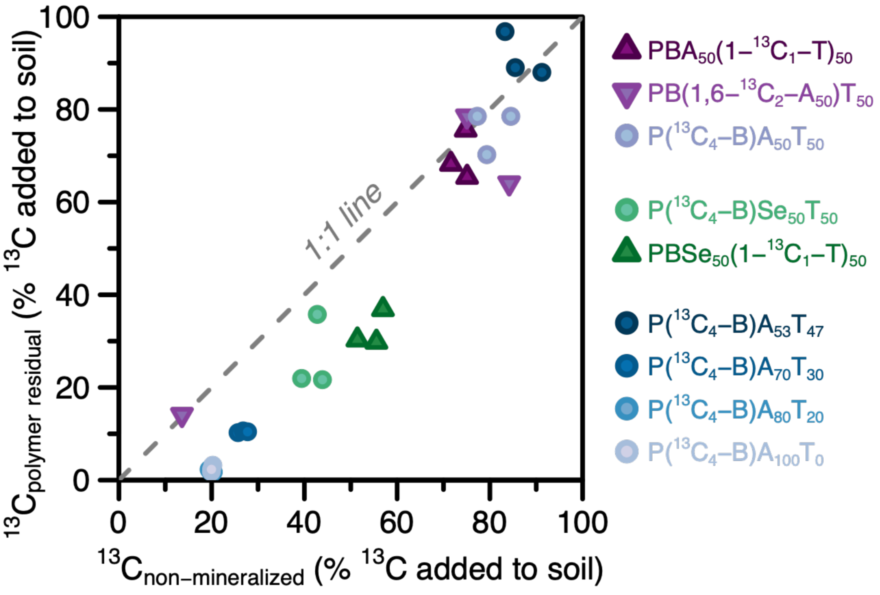

Figure S4. Correlation between residual polyester ($^{13}C_{polymer\ residual}$) and the total non-mineralized polyester-^{13}C ($^{13}C_{non\text{-}mineralized}$) at the end of soil incubations. Values are also shown in **Figure 3** of the main text. Points are shown for individual triplicate incubations of each polyester variant. The dashed grey line indicates a 1:1 relationship.

Section S9. Interpretation of $^{13}C_{biomass}$

The quantification of both $^{13}C_{non\text{-}mineralized}$ and $^{13}C_{polymer\ residual}$ allows estimating the apparent extent to which polyester-derived ^{13}C was incorporated into microbial biomass, $^{13}C_{biomass}$, during biodegradation (i.e., $^{13}C_{biomass} = {}^{13}C_{non\text{-}mineralized} - {}^{13}C_{polymer\ residual}$). However, this assessment needs careful interpretation for several reasons. First, the incubation times and thus the times at which soils were analyzed varied across experiments (i.e., incubation times of 145, 319, and 425 days). Second, the polyesters remaining in the soils were analyzed only at the final time of each incubation (and not over a biodegradation time series via sacrificial sampling of single replicates per monomer-specific labelled variants of PBAT and PBSeT). Therefore, the dynamics of $^{13}C_{biomass}$ throughout polyester biodegradation were not assessed (and it is conceivable that part of the formed $^{13}C_{biomass}$ had mineralized by the time that the incubations were terminated and the soil analyzed). Third, the extent of ^{13}C incorporation into biomass is likely monomer specific and, for a given monomer, even dependent on the position of the carbon in the monomeric unit.[2] Non-labelled carbon atoms in the tested polyesters may thus show slightly different extents of incorporation into biomass as compared to the ^{13}C. Finally, we note that $^{13}C_{mineralized}$ includes not only polyester-derived ^{13}C which is directly mineralized during microbial utilization of polyester-derived carbon, but also polyester-derived ^{13}C that first was incorporated into microbial biomass but then mineralized to $^{13}CO_2$ in the process of biomass turnover. The $^{13}CO_2$ stemming from biomass turnover cannot be delineated from $^{13}CO_2$ directly formed through substrate mineralization. Such polyester-derived ^{13}C first incorporated into biomass but then mineralized would not contribute to the measured pool of $^{13}C_{biomass}$. It is thus possible that we underestimated $^{13}C_{biomass}$ using our approach, especially in the case that the rate of biomass-^{13}C turnover and mineralization to $^{13}CO_2$ is on par with (or even faster than) that of direct polyester carbon

mineralization. In the future, further insights into $C_{biomass}$ dynamics and carbon use efficiencies of polyester carbon can be obtained by characterizing the non-mineralized carbon at different time-points throughout the biodegradation process.

S10. References

(1) Zumstein, M. T.; Schintlmeister, A.; Nelson, T. F.; Baumgartner, R.; Woebken, D.; Wagner, M.; Kohler, H.-P. E.; McNeill, K.; Sander, M. Biodegradation of Synthetic Polymers in Soils: Tracking Carbon into CO_2 and Microbial Biomass. *Sci. Adv.* **2018**, *4*, eaas9024. https://doi.org/10.1126/sciadv.aas9024.

(2) Nelson, T. F.; Baumgartner, R.; Jaggi, M.; Bernasconi, S. M.; Battagliarin, G.; Sinkel, C.; Künkel, A.; Kohler, H.-P. E.; McNeill, K.; Sander, M. Biodegradation of Poly(Butylene Succinate) in Soil Laboratory Incubations Assessed by Stable Carbon Isotope Labelling. *Nat. Commun.* **2022**, *13* (1), 5691. https://doi.org/10.1038/s41467-022-33064-8.

(3) Staddon, P. L. Carbon Isotopes in Functional Soil Ecology. *Trends Ecol. Evol.* **2004**, *19* (3), 148–154. https://doi.org/10.1016/j.tree.2003.12.003.

(4) Nelson, T. F.; Remke, S. C.; Kohler, H. P. E.; McNeill, K.; Sander, M. Quantification of Synthetic Polyesters from Biodegradable Mulch Films in Soils. *Environ. Sci. Technol.* **2020**, *54*, 266–275. https://doi.org/10.1021/acs.est.9b05863.
